# T cells with increased responsiveness cause obesity in mice without diet intervention

**DOI:** 10.1016/j.isci.2024.109471

**Published:** 2024-03-11

**Authors:** Ida Gregersen, Xiang Y. Kong, Sander Kooijman, Håvard Foyn, Helene Grannes, Maria B. Olsen, Anna M. Lone, Kuan Yang, Ana Quiles-Jiménez, Marianne Tran, Jonas Øgaard, Filip M. Segers, Azita Rashidi, Ellen Lund Sagen, Knut H. Lauritzen, Amanda C.M. Pronk, Jan Freark de Boer, Kirsten B. Holven, Espen Melum, Pål Aukrust, Kjetil Taskén, Sverre Holm, Patrick C.N. Rensen, Tuva B. Dahl, Bente Halvorsen

**Affiliations:** 1 Research Institute of Internal Medicine, Oslo University Hospital Rikshospitalet, Sognsvannsveien 20, 0372 Oslo, Norway; 2 Institute of Clinical Medicine, Faculty of Medicine, University of Oslo, Oslo, Norway; 3 Department of Medicine, Division of Endocrinology, Leiden University Medical Center, Leiden, the Netherlands; 4 Einthoven Laboratory for Experimental Vascular Medicine, Leiden University Medical Center, Leiden, the Netherlands; 5 Department of Cancer Immunology, Institute of Cancer Research, Oslo University Hospital, 0424 Oslo, Norway; 6 K.G. Jebsen Centre for B Cell Malignancies, Institute of Clinical Medicine, University of Oslo, 0317 Oslo, Norway; 7 Department of Research and Development, Division of Emergencies and Critical Care, Oslo University Hospital HF, Rikshospitalet, Oslo, Norway; 8 University Medical Center Groningen, Department of Pediatrics, Section Molecular Metabolism & Nutrition, Department of Laboratory Medicine, Groningen, the Netherlands; 9 Department of Nutrition, Institute of Basic Medical Sciences, University of Oslo, Oslo, Norway; 10 Norwegian National Advisory Unit on Familial Hypercholesterolemia, Oslo University Hospital, Oslo, Norway; 11 Norwegian PSC Research Center, Department of Transplantation Medicine, Division of Surgery, Inflammatory Diseases and Transplantation, Oslo University Hospital Rikshospitalet, Oslo, Norway; 12 Section of Gastroenterology, Department of Transplantation Medicine, Division of Surgery, Inflammatory Diseases and Transplantation, Oslo University Hospital Rikshospitalet, Oslo, Norway; 13 Hybrid Technology Hub-Centre of Excellence, Institute of Basic Medical Sciences, Faculty of Medicine, University of Oslo, Oslo, Norway; 14 Division of Cardiovascular Medicine, Department of Medicine, Brigham and Women’s Hospital, Harvard Medical School, Boston, MA, USA

**Keywords:** Immunology, Nutrition

## Abstract

Obesity is a complex multicausal disease that can cause morbidity and mortality, and there is need for improved knowledge on the underlying mechanisms. Using a mouse model of increased T cell responsiveness, we show that development of obesity can be driven by immune cells. This was confirmed with bone marrow transplantation and adoptive T cell transfer to several recipient mouse models. Single-cell RNA sequencing and CyTOF analysis showed that the mice display altered composition of circulating T cells and increased T cell activation in visceral adipose tissue, suggesting activated T cells as critical players in the increased fat mass. In this study, we provide evidence that obesity can be driven by immune cell activity and in particular by T cells, which could have broad implications for prevention and treatment of this condition.

## Highlights

• Mice with increased T cell responsiveness develop obesity, without diet intervention
• The obesity phenotype can be transferred by immune cells to several recipient models
• Recipient mice display increased inflammation in visceral adipose tissue

## Graphical abstract

**Figure:**
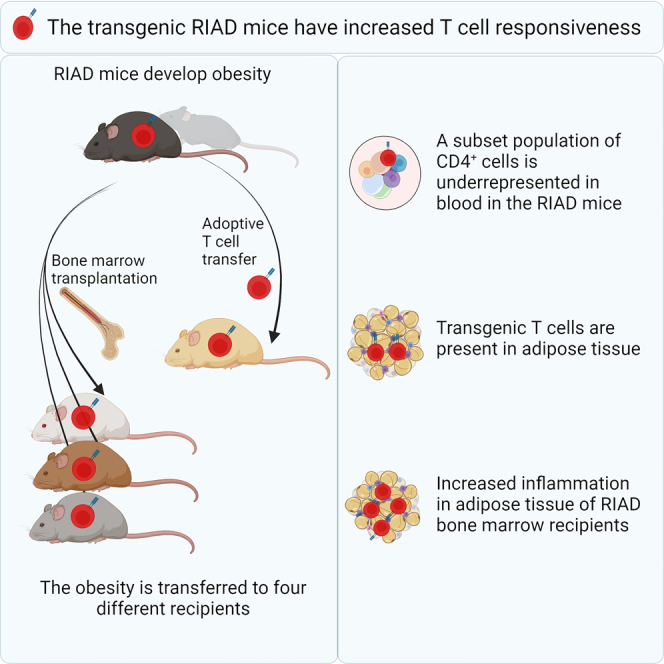

## Teaser

Immunology; Nutrition; Obesity

## Introduction

Obesity is no longer a problem found mainly in high income countries; the global prevalence of obesity now surpasses the prevalence of underweight. Adult obesity significantly increases the risk associated with related disorders, such as type 2 diabetes and various cardiovascular diseases, which is the number one cause of death in the world.^1^^,^^2^ Recent studies suggest that in essence, all immune cells contribute to obesity-related inflammation and immune activation. A recent single-cell RNA sequencing (scRNA-seq) analysis revealed 28 distinct cell populations in human white adipose tissue (WAT), of which 15 were immune cell subsets.^3^ Of these, there was an increased proportion of dendritic cells (DCs), unconventional T cells, innate lymphoid cells (ILCs), and myeloid-like and natural killer (NK)-like cells in WAT samples from individuals with obesity compared to WAT from lean individuals^3^ However, even though adipose tissue immune cell infiltration and activation are known to be important components in the pathogenesis of obesity and obesity-related disease,^4^ the mechanisms underlying the role of immunity in obesity development *per se* are not known. One notable exception is that CD4^+^ T cells have been shown to memorize obesity and promote weight regain in a mouse model, supporting a causal role of T cells in the development of adiposity.^5^ Herein, we will further explore the causal role of T cells in obesity development, through several experimental approaches.

The type I protein kinase A (PKA I)/cAMP pathway plays a crucial role in T cell activation, by functioning as a negative control mechanism,^6^ which represents a potentially important checkpoint for obesity development driven by T cells. In the current study, we explore this hypothesis by using a mouse model expressing a soluble RI anchoring disruptor (RIAD) which specifically displaces PKA I from A-kinase anchoring proteins in T and NK cells, thereby releasing the tonic inhibition of immune activation in these cells, mediated by hormones and anti-inflammatory mediators signaling through cAMP. Increased T cell responsiveness^7^ has previously been confirmed in these mice, and here we show that this alteration in T cell activity leads to obesity development. These findings reveal novel mechanisms in obesity development and underscore the potential role of immune cells, and T cells in particular, as drivers of this process.

## Results

### Mice with enhanced T cell responsiveness develop obesity

**Figure 1:**
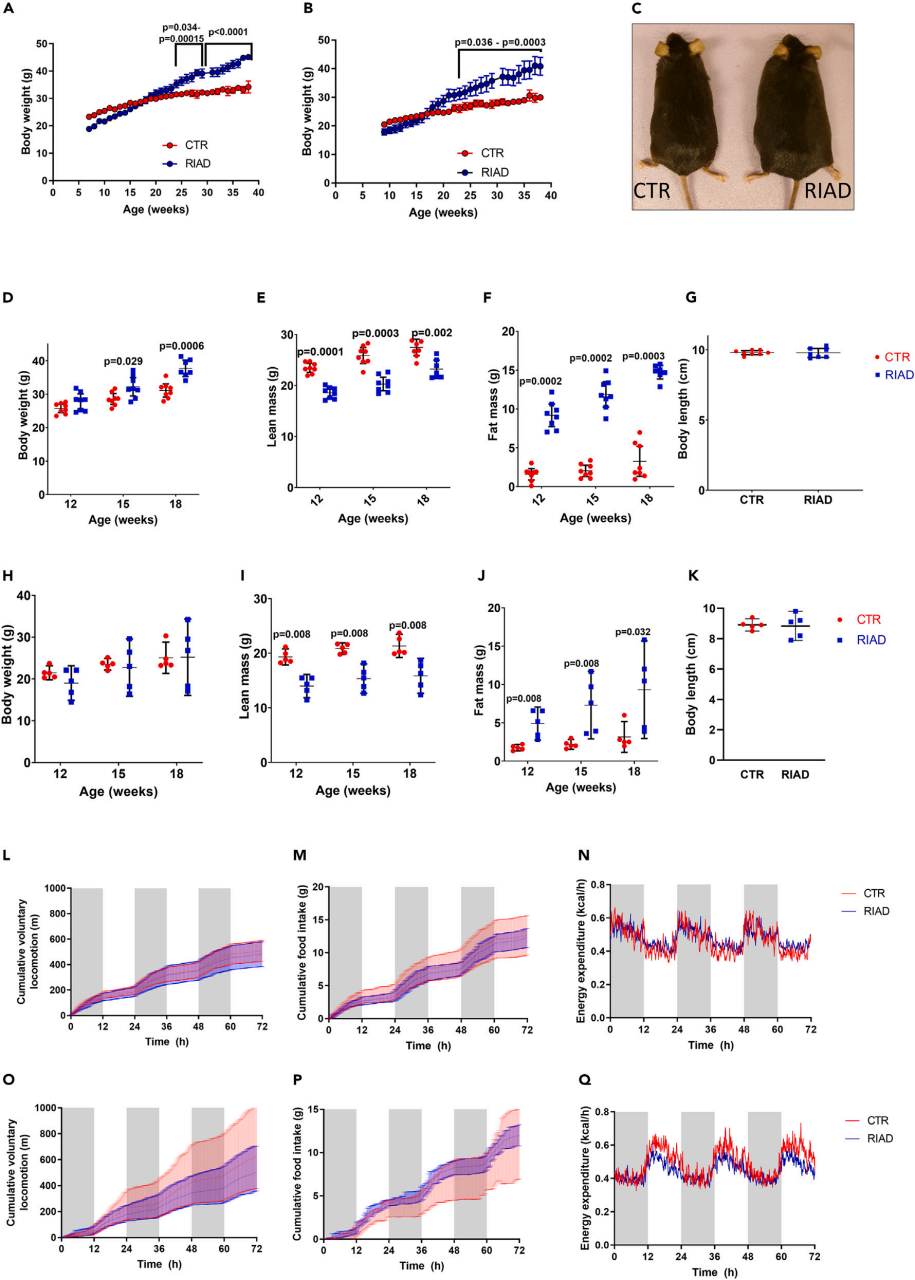
Mice with increased T cell responsiveness develop obesity explained by increased fat mass without high-fat diet intervention Body weight development in (A) male and (B) female control mice (red, n = 4 and 6) and RIAD mice (blue, n = 9 + 5), (C) illustration photo of 40-week-old mice. Body composition was monitored in male mice, and (D) body weight, (E) lean body mass and (F) fat mass was determined at 12, 15, and 18 weeks of age. (G) Illustrates body length of male mice at 18 weeks of age. Body composition of female mice: (H) body weight, (I) lean body mass and (J) fat mass, determined at 12, 15, and 18 weeks of age. (K) Illustrates body length of female mice at 18 weeks of age. The mice were monitored in metabolic cages and cumulative movement, cumulative food intake and energy expenditure was assessed during a period of 72 h, (L–N) male mice, (O–Q) female mice. n = 5–8, data analyzed with t-test or Mann-Whitney test and presented as mean and 95% CI.

To explore whether increased T cell responsiveness could affect body weight, we followed our transgenic RIAD model^7^ for up to 40 weeks with weakly weighing. Strikingly, when feeding on a normal chow diet and without externally triggering of the immune cells, these mice developed significant obesity compared to their littermate controls. The difference in body weight was evident in both male and female mice from around 20 weeks of age, and increased over time, with the transgenic mice weighing over 30% more at 40 weeks of age (Figures 1A–1C).

To explore the phenotype further we performed a thorough screening in a separate cohort of mice, following them from 12 to 18 weeks of age. Interestingly, EchoMRI analysis of body composition revealed a pronounced increase in body fat and lower lean body mass at 12 weeks of age, even before any differences in body weight were observed (Figures 1D–1K). All adipose tissue compartments were increased in weight, in male mice, and the same trend was seen in female mice. Also liver weight was increased in male, but not in female mice; and muscle weight was reduced in both genders, but less pronounced in females (Table S1). In contrast, we did not observe any significant alterations in locomotion, energy intake or energy expenditure (Figures 1L–1Q), and only minor differences in plasma lipids between the genotypes (Figure S1). Further experiments found no differences in fecal energy excretion between the genotypes (Figure S2), and the mice also had similar glucose clearance, as shown in an oral glucose tolerance test (OGTT, Figure S3). Furthermore, there were no differences between the genotypes in absolute number of total leukocytes, lymphocytes, monocytes or granulocytes, as measured by differential blood count (Table S2).

### Reduced lipolysis in RIAD adipose tissue

To further explore the mechanisms underlying the observed obesity in our model, we performed an *ex vivo* lipolysis assay. WAT explants from transgenic RIAD mice released less glycerol and non-esterified free fatty acids (NEFA) as compared to adipose tissue from control mice under β-adrenergic-stimulated (isoproterenol) conditions, suggesting reduced lipolysis in adipose tissue of RIAD mice (Figure S4).

### A population of circulating CD4^+^ T cells is underrepresented in RIAD mice

**Figure 2:**
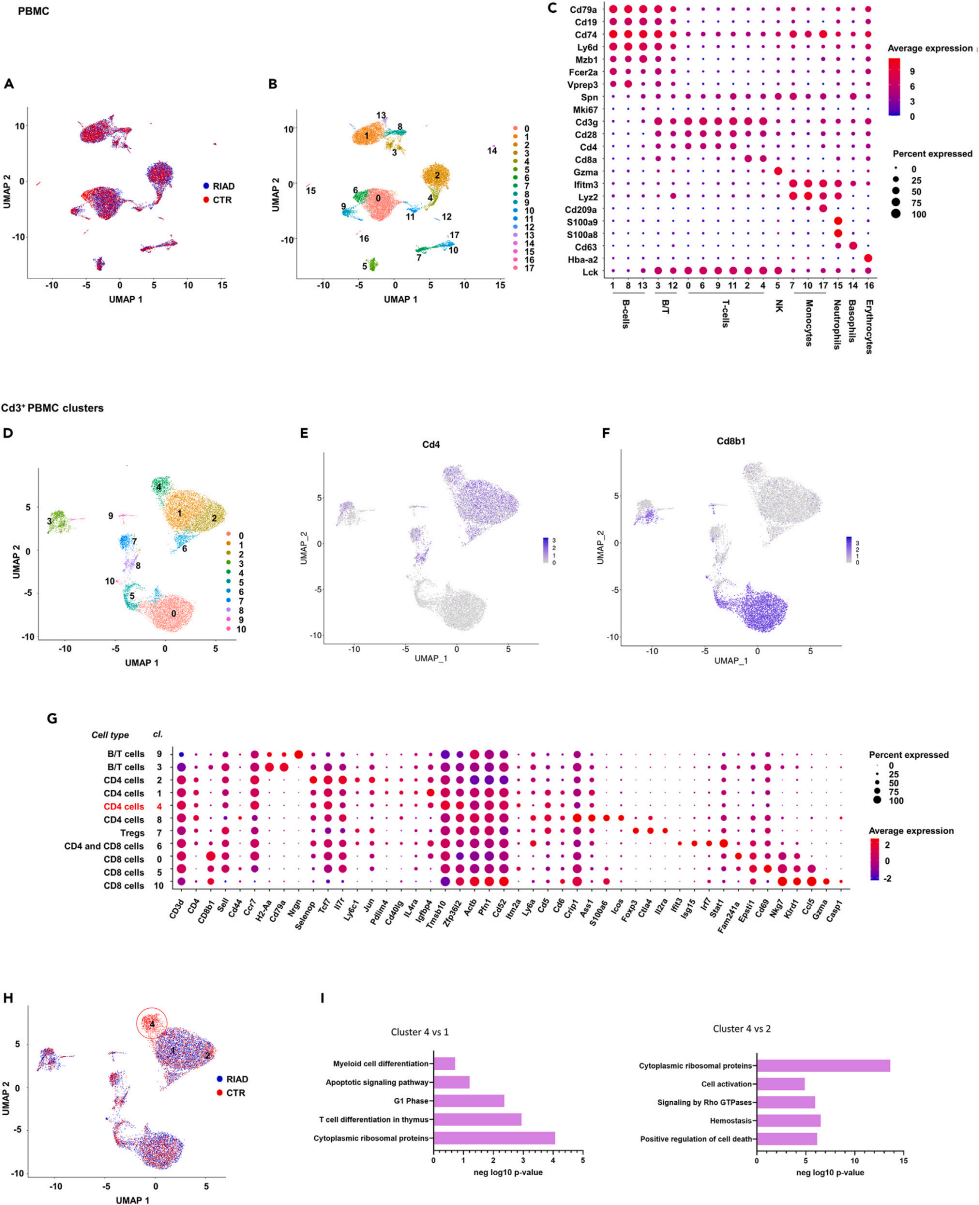
Single-cell sequencing analysis of PBMCs reveals a single population of circulating CD4^+^ T cells to be underrepresented in transgenic mice Uniform manifold approximation and projection (UMAP) of all sequenced cells by (A) genotype and (B) cluster. (C) Marker genes for each cluster. Cells from CD3^+^ cluster 3, 12, 0, 6, 9, 11, 2 and 4 were reclustered and (D) UMAP showing cells in 11 new clusters is shown, together with their (E) CD4 expression and (F) CD8 expression. (G) Marker genes for each cluster. (H) UMAP showing cells marked by genotype. Cluster 4, underrepresented in RIAD, is encircled. (I) Gene enrichment analysis including differentially expressed genes (DEGs) comparing cluster 1 vs. cluster 4 (left panel) and cluster 2 vs. cluster 4 (right panel). N = 1 + 1.

In order to characterize the RIAD immune cell phenotype, we performed scRNA-seq analysis of peripheral blood mononuclear cells (PBMCs) of two months old mice. This analysis was performed at an early time point to potentially identify immune regulatory mechanisms preceding the increase in body weight, and thus to prevent that an established obesity could confound the data. We performed unsupervised community detection to cluster the data based on highly variable genes (File S1), and cells were projected in two dimensions using Uniform Manifold Approximation and Projection (UMAP; Figure 2A). 15 different PBMC subpopulations were detected, of which six were T cell populations, three were B cell populations, two populations contained both T- and B cell markers, three were monocyte populations and one was an NK cell population (Figures 2B and 2C). RIAD and control cells showed overlapping clustering in most cell populations, but cluster differently within two CD4^+^ cell populations, cluster 9 and 6.

To further explore the distribution of T cells, all CD3^+^ clusters were subjected to re-clustering, identifying 11 sub-clusters (Figures 2D–2G, File S2). One cluster (CD4^+^ T cell cluster 4) was highly overrepresented in control (13.34% of all clustered cells) compared to RIAD cells (0.08% of all clustered cells, Figure 2H). The marker genes of cluster 4 showed great overlap with CD4^+^ T cell cluster 1 and 2, which was similarly distributed among the genotypes (Figure 2G). The differently expressed genes (DEGs) separating these clusters (File S3), constituted terms and pathways related to cell development and activation status, i.e., “myeloid cell differentiation”, G1 phase”, and “T cell differentiation in thymus” (cluster 4 vs. cluster 1, Figure 2I); and “cytoplasmic ribosomal proteins”, “cell activation” and “positive regulation of cell death” (cluster 4 vs. cluster 2, Figure 2I), suggesting that the CD4^+^ T cells of cluster 4, underrepresented in RIAD, have an altered activation status compared to cells in cluster 1 and 2, which were represented in both genotypes.

### Increased T cell activation in adipose tissue of RIAD mice

**Figure 3:**
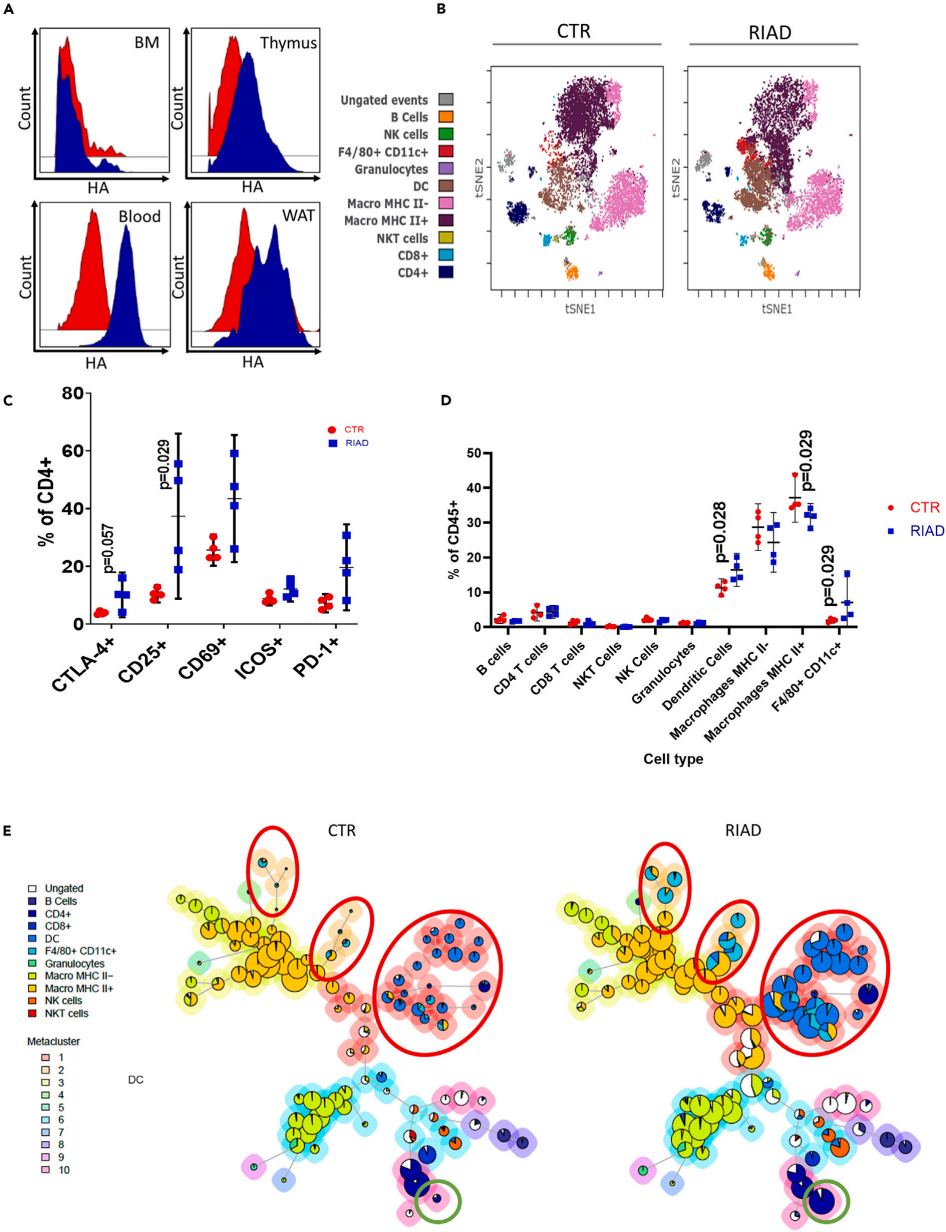
More activated and senescent T cells in white adipose tissue of transgenic mice (A) HA-tag abundance in CD3^+^ cells from bone marrow (BM), thymus, blood and white adipose tissue (WAT) from control (red) and RIAD (blue) mice. (B) Representative viSNE plots of CD45^+^ events of cells harvested from Control and RIAD WAT (n = 4 + 4). Equal number of events in each plot. Plots are overlaid with colors representing the type of cells identified through manual gating. DCs are defined as CD11c positive cells and macrophages as F4/80 positive cells. Macrophages were split based on their expression of MHC II. In addition, an F4/80+CD11c+ population was identified that were mainly present in RIAD. (C) T cell activation and exhaustion markers. Gating for CD4^+^ and CD8^+^ T cells positive for activation or exhaustion markers (D) Overview of cell type abundance. n = 4 + 4, data analyzed with Mann-Whitney test and presented as mean and 95% CI (a,c,d). (E) FlowSOM clustering. Plots demonstrate higher abundance (bigger nodes) of dendritic cells (Blue) and F4/80 and CD11c double-positive (light blue) cells marked by red circles in RIAD compared to control. The node highlighted by the green circle represents a CD4^+^ population with higher expression of CD25 and CD69.

Our findings so far suggest only minor differences in circulating immune cells in the transgenic mice, but with a population of CD4^+^ T cells (CD4 positive T cell cluster 4) underrepresented among circulating cells in RIAD mice. To explore the actual scene of event in obesity development, we looked closer into the adipose tissue of the mice. When exploring the presence of “RIAD cells”, measured as the proportion of CD3^+^ cells carrying the HA epitope tag, which is inserted with the RIAD transcript as a positive control, we detected a high level in visceral white adipose tissue (WAT; Figure 3A). This suggests that in the RIAD model, the altered T cells migrate to adipose tissue where they potentially promote the development of adiposity. To explore in detail the T cell phenotype in RIAD adipose tissue, we next performed an extensive mass cytometry analysis (CyTOF) of visceral WAT from RIAD mice with established obesity, and from control mice (Figure S5; Table S3). These analyses showed an increased percentage of CD4^+^ T cells expressing the activation marker CD25 (Il2rα). Further, although not statistically significant, percentage of CD4^+^ T cells expressing the activation markers CD69 and the check-point regulators CTLA-4 and PD-1, also showed a tendency to be increased in the RIAD mice, suggesting increased T cell activation as well as senescent/exhausted T effector cells in the adipose tissue of RIAD mice as compared to control mice (Figures 3B, 3C, and S6). These analyses also revealed increased levels of DCs and F4/80^+^ CD11c^+^ macrophages in WAT from RIAD mice, while there was a small reduction in levels of MHC II^+^ macrophages compared to controls (Figures 3D, 3E, and S6). There were no differences between the genotypes when comparing levels of other immune cells, including CD4^+^ or CD8^+^ positive T cells; or percentage of T regulatory cells (Figures 3D and S7).

### Bone marrow transplantation confirms immune cell-driven obesity in RIAD mice

**Figure 4:**
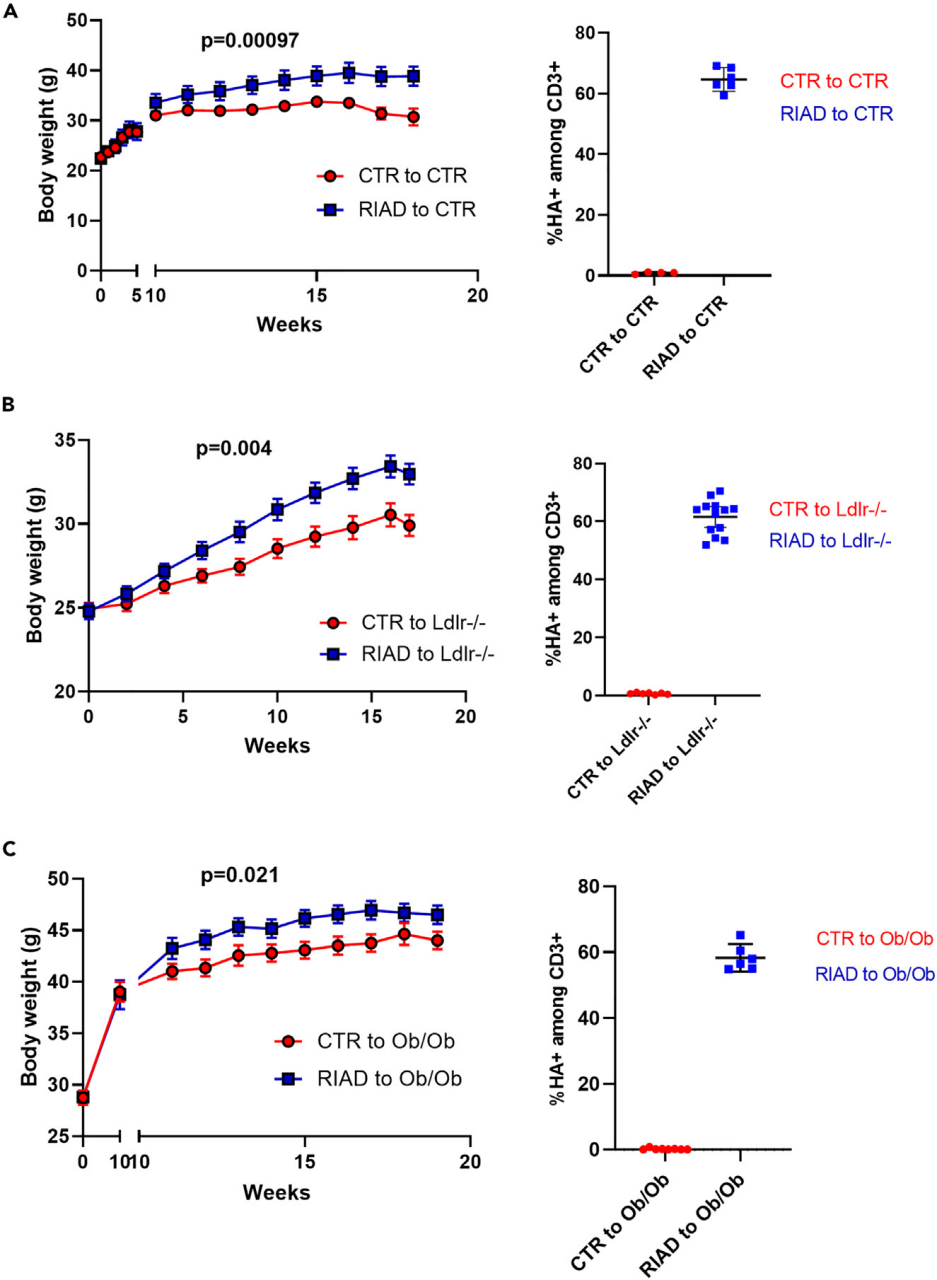
Bone marrow transplantation confirms immune cell-driven obesity in transgenic mice Body weight development in (A) wild type mice, (B) *Ldlr−/−* mice and (C) *Ob/Ob* mice transplanted with bone marrow from control mice (red) or transgenic mice (blue). n = 4–12, p value indicates difference between AUC curves, data presented as mean and SEM. In the panels to the right is the verification of transplantation by HA + CD3^+^ cells in blood in the respective models. Ctr, control; g, grams.

The only inherent difference between RIAD mice and control mice is T cell responsiveness, suggesting that immune cells are the drivers of obesity in this model. To explore the causal relationship between these cells and the development of obesity, we performed bone marrow transfer experiments where bone marrow from the RIAD mice was transferred into irradiated wild type mice, as well as *Ob/Ob* mice, an established mouse model of obesity,^8^ and *Ldlr*^−/−^ mice, a mouse model of hypercholesterolemia.^9^ Strikingly, all three groups of recipient mice transplanted with bone marrow from the RIAD mice displayed significantly increased body weight compared to mice transplanted with control bone marrow (Figures 4A–4C). The increases in body weight were 26.5%, 5.7% and 10.2% for WT mice, *Ob*/*Ob* mice and *Ldlr*^−/−^ mice, respectively. The presence of the HA-tag in the circulation of recipient mice after transplantation was confirmed by flow cytometry in all three models (Figures 4A–4C). Further, RIAD cells were present in several different adipose compartments, including epididymal (visceral) WAT, subcutaneous WAT, and BAT (Figure S8), demonstrating infiltration of these cells into these adipose tissues. These data, unequivocally demonstrate that the observed obesity in the RIAD mice is immune cell-driven.

### T cells are the causal drivers of obesity in RIAD mice

**Figure 5:**
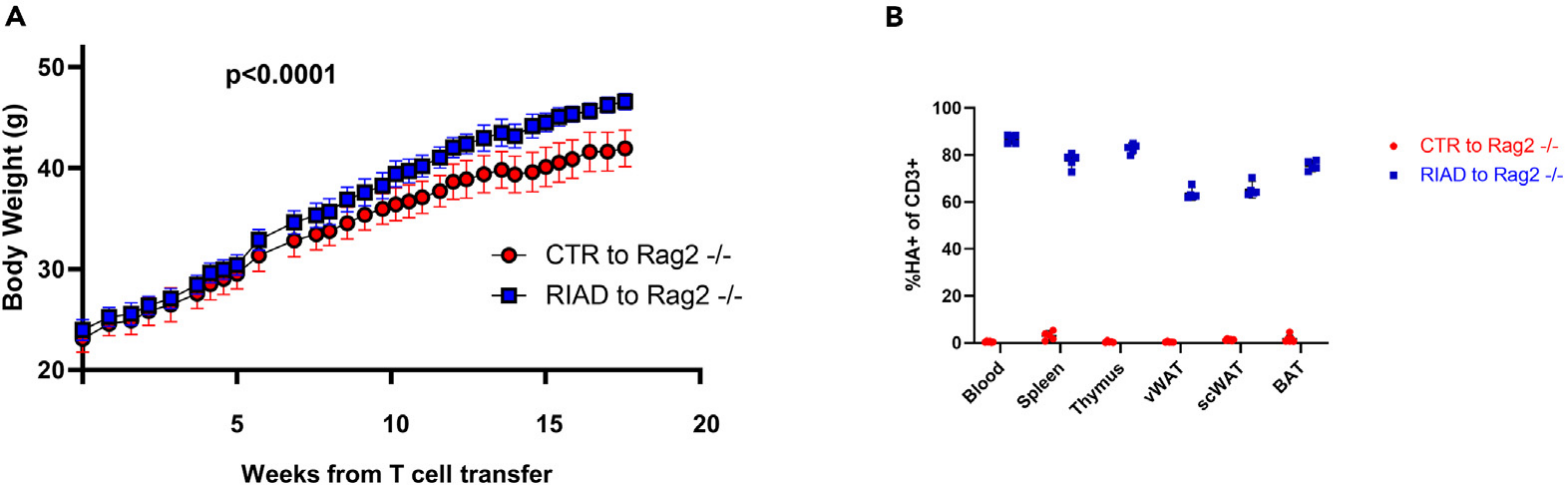
T cells from transgenic mice promote weight gain in Rag2^−/−^ mice Body weight development in Rag2^−/−^ mice after adoptive T cell transfer from transgenic RIAD mice (n = 5) and control mice (n = 6). p value indicates difference between AUC curves, data presented as mean and SEM (A) time curve. (B) Percentage of HA^+^ CD3^+^ cells in blood and tissues in recipient mice after 24 weeks.

To further pinpoint if the altered T cells play a causal role in obesity development in our model, we performed an adoptive T cell experiment. CD3^+^ T cells were isolated from RIAD mice and control mice and transferred to B cell- and T cell-deficient Rag2^−/−^ mice, and body weight was monitored. As shown in Figure 5A, mice receiving T cells from RIAD mice developed significantly higher body weight as compared to mice receiving T cells from control mice. The presence of the HA-tag in the circulation and in the adipose tissue of recipient mice was confirmed by flow cytometry at harvest, 24 weeks after the T cell transfer (Figure 5B).

### RNA sequencing indicates altered immune cell composition and inflammatory processes in adipose tissue of RIAD bone marrow recipients

**Figure 6:**
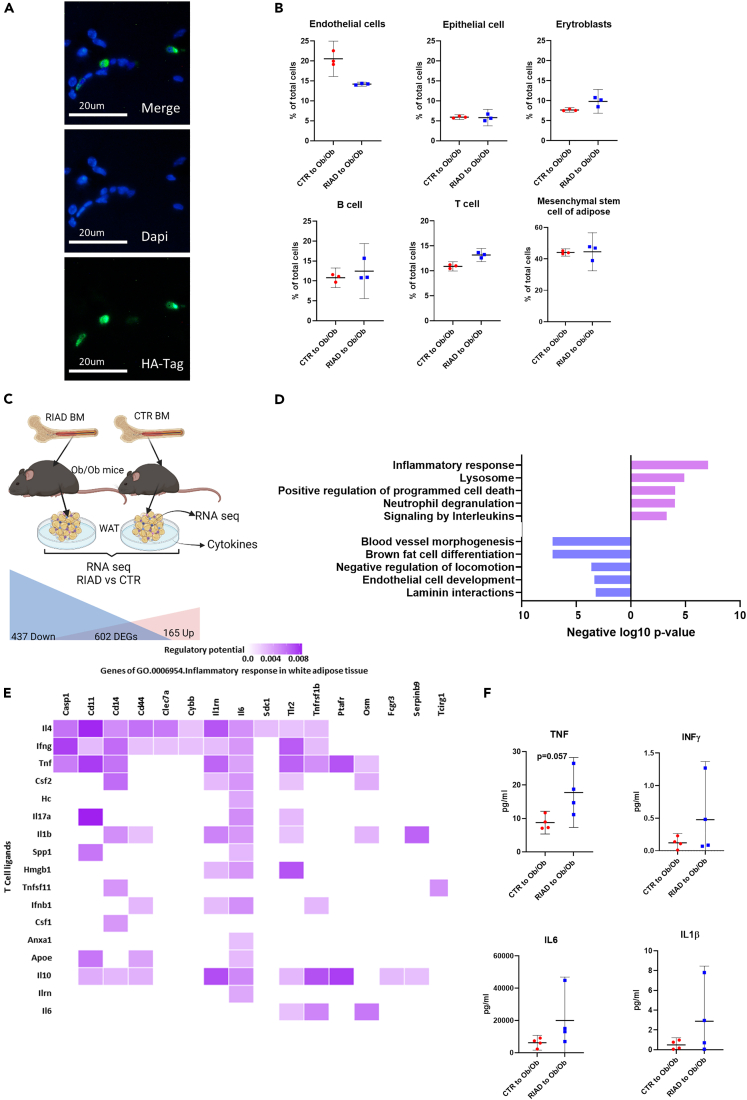
Adipose tissue from mice transplanted with bone marrow of transgenic mice display increased inflammation Epididymal adipose tissue from bone marrow transplanted *Ob/Ob* mice receiving bone marrow (BM) from RIAD (RIAD, n = 3)) or control (control, n = 3) mice were cultured over-night. Gene expression was mapped by RNA sequencing and secretion of cytokines was measuring in medium. (A) Immunofluorescent staining of HA-tag in PG from *Ob/Ob* mice receiving RIAD BM. (B) Cell abundance estimated by CIBERSORT digital cytometry. Data analyzed with Mann-Whitney test and presented as mean and 95% CI. (C) Schematic overview of the experimental setup and number of DEGs in the adipose tissue. (D) Gene enrichment analysis showing top five enriched terms and pathways including differentially expressed genes (DEGs) up-regulated or down-regulated in RIAD vs. control. (E) Regulatory potential for T cell ligand candidates for DEGs involved in *GO 0006854 Inflammatory response,* predicted by NicheNetr R package (Browaeys et al., 2020). (F) Level of TNF, IFNγ, IL6 and IL1β in the adipose tissue media 24 h after tissue explant. Data analyzed with Mann-Whitney test and presented as mean and 95% CI.

The genetically modified T cells are present in the adipose tissue of RIAD mice (Figure 3A) and this was also true for the recipients of RIAD T cells and bone marrow (Figures 5, 6A, and S8, respectively). To explore the adipose tissue of the bone marrow recipients further, we performed RNA sequencing (RNA-seq) analysis of visceral and subcutaneous adipose tissue from transplanted *Ob/Ob* mice. Digital cytometry (Cibersort^10^) showed no difference in estimated abundance of adipocyte mesenchymal stem cells (Figure 6B), between the genotypes, suggesting that the adipose tissue growth is driven by adipocyte hypertrophy, as also suggested by increased adipocyte size in visceral adipose tissue of RIAD mice (Figure S9). Further, visceral adipose tissue from RIAD bone marrow-recipients displayed slightly, although not significantly, more T cells, which could indicate infiltration or local proliferation of T cells in the visceral adipose tissue of recipient mice and further suggest these cells as drivers of adiposity (Figure 6B). There were no differences in immune cell composition of the subcutaneous fat (Figure S10). At the transcriptional level in visceral adipose tissue, we found 602 DEGs in mice transplanted with RIAD compared to control bone marrow, where the majority (437 genes) were down-regulated and enriched in terms and pathways supporting decreased vascularization and altered differentiation of brown adipocytes (Figures 6C and 6D, File S4). Further, 165 genes were up-regulated in the RIAD-transplanted mice and these genes were enriched in terms and pathways related to inflammatory processes (Figures 6C and 6D, File S4).

We next used NicheNetr R package to predict the T cell expressed ligands and adipose tissue receptors interactions that might drive these gene expression changes in the adipose tissue.^11^ In this analysis, gene transcripts detected by RNA seq of CD3^+^ splenic RIAD T cells were considered as potential ligand candidates (File S5). Regulatory potential for the ligand candidates was tested on the DEGs found in terms/pathways shown in Figure 6D. As shown in Figure 6E and File S5, several potential interactions were found. Of the identified ligands which could potentially regulate the inflammatory response in the adipose tissue, we chose TNF, INFγ, IL6 and IL1β based on their roles in regulation of metabolic processes, and their levels were measured in the adipose tissue media 24 h after tissue explantation. As depicted in Figure 6F, adipose tissue of RIAD-transplanted mice released more, although not statistically significant, TNF as compared to control-transplanted mice (p = 0.06).

## Discussion

Herein we present a model for T cell-driven obesity, revealing new knowledge about the role of immune cells in obesity development. Mice with an altered T cell signaling (i.e., attenuated tonic inhibition of T cell activation through the cAMP/PKAI pathway, termed RIAD), resulting in T cells with increased responsiveness, display adiposity at an early age and develop obesity without diet intervention or external triggering of the immune system. Importantly, we show that bone marrow transplantation from RIAD mice is sufficient to drive the development of obesity in recipient mice of various genotypes (i.e., WT, *Ob*/*Ob* and *Ldlr*^−/−^ mice); and that adoptive T cell transfer from RIAD mice increase body weight in T cell-deficient mice; illustrating the strong potency of T cells to promote obesity. The presence of the altered T cells in the adipose tissue of RIAD bone marrow and T cell recipients, support local effects of T cells on development of adiposity. Further, we demonstrated that visceral WAT from RIAD mice are enriched in activated and exhausted/senescent T cells suggesting these as potential mediators of the obesity phenotype and provides important new knowledge about the causal role of immune cells in obesity development.

The role of immune cells in mediating weight gain is not clear; however, weight cycling has been shown to induce T cell accumulation in epididymal adipose tissue.^12^ Further, Zou et al. showed that obesity memory and weight regain in mice were largely related to CD4^+^ T cell activation, supporting a role for immune cells as mediators of obesity *per se*, and not just as mediators in obesity-induced inflammation.^5^ The present study is however, the first to show that T cells with increased responsiveness, by attenuating cAMP/PKAI-mediated T cell inhibition, promote obesity. The obesity in our model is accompanied by the presence of activated T cells in WAT, suggesting local stimulation of adiposity. Development of obesity was not only observed in the transgenic mouse model, but also in mice of three different genotypes that received bone marrow cells from RIAD mice, as well as in mice only receiving T cells from the RIAD mice. These collective data strongly suggest a causal link between T cell activation and obesity. In humans, sCD25 is positively associated with waist circumference, a marker of abdominal fat mass, supporting a role for activated T cells also in human obesity.^13^ Nonetheless, any causal role of T cells in human obesity is yet to be determined.

scRNA-seq analyses of circulating PBMCs showed only minor alterations in RIAD mice, underscoring that the activation of immune cells occurs primarily within adipose tissue. We found, nevertheless, some evidence of a CD4^+^ T cell subpopulation that was nearly absent in RIAD mice. The analyses were performed early (2 months of age) before any overt obesity, and we cannot exclude that the modest differences between the genotypes could at least partly reflect this. Further, even though there were no significant body weight at this age, the mice displayed increased adiposity, and thus we cannot exclude that this can confound our findings. Nevertheless, we suggest that in the transgenic model, the cells of this identified subpopulation are activated and have migrated to the adipose tissue and are therefore underrepresented in the circulation. Indeed, within WAT of the RIAD mice, T cells showed a phenotype characterized by inflammation and immune exhaustion/cellular senescence, suggesting that activated T cells migrate to WAT and stimulate the expansion of the fat mass in these mice. Senescent T cells are inflammatory and known drivers of adipose tissue inflammation during obesity.^14^ Although not significant, we saw a tendency toward increased numbers of T cells also within visceral adipose tissue of mice that had undergone bone marrow transplantation or adoptive T cell transfer from RIAD mice. This inflammatory T cell-driven microenvironment could in turn lead to the recruitment of inflammatory DCs and monocyte-derived macrophages into WAT as a secondary phenomenon. In fact, we found increased levels of these myeloid cells, both DCs and F480 CD11c positive macrophages, in WAT of RIAD mice. Infiltration of inflammatory T cells to adipose tissue has previously been shown to precede the accumulation of macrophages,^15^ supporting this hypothesis.

On the other hand, Cd11c positive cells have been shown to be important in the accumulation and activation of T cells in adipose tissue,^16^ As the adipose tissue of RIAD mice also displayed increased numbers of these cells, they could potentially further increase the presence and activation of T cells in our model. Moreover, whereas T cell activation induces activation of surrounding macrophages and DCs within the WAT microenvironment, these cells, in turn, further activate T cells through the release of inflammatory cytokines. Hence, the lack of tonic inhibition of cAMP/PKAI-mediated T cell activation in the transgenic mouse model seems to promote a bidirectional pathogenic inflammatory loop within WAT leading to obesity. Conversely, DCs have also been shown to control adipose tissue homeostasis under steady-state conditions, preventing uncontrolled adipose tissue expansion.^17^ We speculate that the infiltration of DCs into the adipose tissue in our model could also be a compensatory mechanism to counteract the increased adiposity, but this needs further investigation.

We saw a tendency toward increased levels of PD-1^+^CD4^+^ T cells in WAT of RIAD mice. PD-1, like CTLA-4, is a senescence marker of T cells, and PD-1^+^ T cells accumulate in WAT in high-fat diet-induced obesity.^18^ Further, senescent T cells are shown to induce BAT whitening, which is involves adipose inflammation and adipose tissue remodeling.^19^ Pathway analysis suggests reduced browning in WAT from *Ob*/*Ob* mice transplanted with RIAD bone marrow, supporting this association, and providing an additional mechanistic route for expansion of the WAT through T cell signaling in our model. Senescent immune cells are also known to release increased levels of TNF as compared to normal cells,^20^ and indeed we found elevated, although not statistically significant, TNF levels secreted from visceral adipose tissue of RIAD transplanted *Ob/Ob* mice. Our ligand/receptor interaction analyses suggest that this inflammatory cytokine could be a local mediator of T cell-driven inflammation of WAT in these mice, however this needs further investigation.

The RIAD mice express a peptide that halts the cAMP-PKA I pathway. In response to cAMP, induced by prostaglandins (PGs), adrenergic stimuli, inflammatory mediators and several other ligands, this pathway attenuates proximal T cell activation.^6^ Thus, the transgenic T cells, with disrupted PKA I signaling, are not by nature more active, but more responsive, as the signaling cascade upon activation is not turned off. What activates the cells in our model is at present not clear. Yet, our data suggest that the cells are primarily activated within WAT. Inflammatory mediators from adjacent macrophages and DCs are likely contributors, but also β-adrenergic signaling could be of importance, since β2 adrenergic receptors are found on CD4^+^ and CD8^+^ cells.^21^^,^^22^ WAT is innervated by the sympathetic nervous system and its activation is a principal initiator of lipid mobilization.^23^ In the transgenic mouse model, any adrenergic stimuli of the T cells would result in sustained activation of effector T cells,^21^ that could ultimately lead to reduced lipolysis and favor stimulation of energy storage in adipose tissue.^24^ Therefore, although the development of adiposity was not dependent on high-fat diet or external stimuli; several activation signals could have contributed to the T cell-mediated adiposity in the transgenic mice, including the release of inflammatory molecules and β-adrenergic stimuli within WAT. We found no increase in food intake, energy expenditure, or fecal loss of energy. However, this could be due to difficulties in detecting small differences of these measures, which are highly affected by body weight and composition. It is therefore possible that a minor increase in food intake and/or a minor decrease in energy expenditure, over time could have led to the observed increase in body weight in RIAD mice. Nonetheless, even though the exact mechanisms for increased adiposity in our model are still elusive, we show herein that the differences in body weight is in fact driven by immune cells and in particular T cells. We demonstrate this through several experimental approaches, including bone marrow transplantation and adoptive transfer of T cells from RIAD mice into to recipient mice of various genotypes. Our study provides new insight to the causality of T cells in the pathogenesis of obesity.

### Limitations of the study

The study has some limitations. In some of the experiments, the number of included animals were low, such as in the single cell analyses in peripheral blood. Further, future studies should aim at describing the WAT-specific T cells and how these affect adipogenesis in the transgenic model as well as the exact molecular mechanisms linking T cells to adipogenesis.

## STAR★Methods

### Key resources table

**Table:** 

REAGENT or RESOURCE	SOURCE	IDENTIFIER
**Antibodies**		

Anti-CD3	Macs Miltenyi Biotec	Cat# 130-117-671; RRID:AB_2728017
Anti- CD4	BD Biosciences	Cat# 560181; RRID:AB_1645235
Anti-CD8	BD Biosciences	Cat# 563068; RRID:AB_2687548
Anti-HA	Macs	Cat# **120-002-687**
Anti-Ly-6G	Standard BioTools	Cat# 3141008B; RRID:AB_2814678
Anti-CD185	Standard BioTools	Cat # **3142015B**
Anti-TCRβ	Standard BioTools	3143010B-DVS
Anti-CD69	Standard BioTools	Cat# 3145005B; RRID:AB_2895115
Anti-F4/80	Standard BioTools	Cat# 3146008B; RRID:AB_2895117
Anti-CD45	Standard BioTools	Cat #; 3147003B; RRID:AB_2811243
Anti-CD11b	Standard BioTools	Cat# 3148003B; RRID:AB_2814738
Anti-CD19	Standard BioTools	Cat# 149002B; RRID:AB_2814679
Anti-CD80	Biolegends	Cat# 104735; RRID:AB_2563763
Anti-CD25	Standard BioTools	Cat# 3151007B; RRID:AB_2827880
Anti-CD3ε	Standard BioTools	Cat# 3152004B; RRID:AB_3076460
Anti-HA-Tag	Abcam	Ab256483
Anti-CTLA-4	Standard BioTools	3154008B-DVS
Anti-iNOS	Fisher Scientific	15567456
Anti-CD1d	Biolegends	Cat# 123502; RRID:AB_1236531
Anti-FoxP3	Standard BioTools	Cat# 3158003A; RRID:AB_2814740
Anti-CD279 (PD-1)	Standard BioTools	Cat# 3159023B
Anti-CD62L	Standard BioTools	3160008B-DVS
Anti-Ki-67	Standard BioTools	Cat# 3161007B; RRID:AB_2811255
Anti-Tim-3	Standard BioTools	3162029B
Anti-CD161 (NK1.1)	Standard BioTools	3165018B
Anti-CD335 (Nkp46)	Standard BioTools	Cat# 3167008B; RRID:AB_2922922
Anti-CD8α	Standard BioTools	Cat# 3168003B; RRID:AB_2811241
Anti-CD206	Standard BioTools	Cat# 3169021B; RRID:AB_2832249
Anti-CD169	Standard BioTools	Cat# 3170018B; RRID:AB_2885022-
Anti-CD44	Standard BioTools	Cat# 3171003B; RRID:AB_2895121
Anti-CD4	Standard BioTools	Cat# 3172003B; RRID:AB_2811242
Anti-I-A/I-E (MHC II)	Standard BioTools	Cat# 3174003B; RRID:AB_2922924
Anti-CD127	Standard BioTools	Cat# 3175006B; RRID:AB_2927570
Anti-ICOS	Standard BioTools	Cat# 3176014B
Anti-CD11c	Standard BioTools	Cat# 3209005B; RRID:AB_2811244
Anti-HA-Tag	Cell Signaling	Cat# 3724; RRID:AB_1549585

**Chemicals, peptides, and recombinant proteins**		

EDTA	VWR Chemicals	E522
Type II Collagenase	Sigma	C2-BIOC
Lysis buffer	BioLegend	Cat # 420302
RBC lysis buffer	BioLegend	Cat # 420302
Maxpar staining buffer	Standard BioTools	NC0501752
Cisplatin		
Fc block		
DNA intercalator		
Cell acquisition buffer	Standard BioTools	NC1919529
Element calibration beads	Standard BioTools	NC1307119
penicillin/streptomycin	Gibco	15-140-122
1 % HSA, endotoxin-free	Sigma Aldrich, St. Louis, MI, USA	

**Critical commercial assays**		

Triglycerides enzymatic kit	Roche Diagnostics	Material no. 04657594190
Cholesterol enzymatic kit	Roche Diagnostics	Material no. 07374577001
NEFA-C kit	Wako Diagnostics	Suppl no. 276-76491, 999-34691, 991-34891, 997-76491
Glycerol Assay kit Cell based	Abcam	ab133130
Fixation/Permeabilization kit	BD Biosciences	cat. no. 554714
Free Fatty Acid Fluorometric Assay Kit	Cayman Chemicals	Item no. 700310
T cell isolation kit II	Milteny Biotec	Cat # 130-095-130
Cell-ID 20-Plex Pd Barcoding kit	Standard BioTools	NC0919908
Maxpar X8 Multi-Metal labeling kits	Standard BioTools	NC0245957
Chromium Single Cell 3/ Library & Gel Bead Kit v2	Chromium Controller System, 10X Genomics	
RNeasy Mini kit	Qiagen	Cat. No. / ID: 74104

**Deposited data**		

Sequencing data	NCBI Sequence Read Archive	PRJNA1070442 https://www.ncbi.nlm.nih.gov/bioproject/PRJNA1070442

**Experimental models: Organisms/strains**		

Mouse: C57BL/6 (CTR)	Taconic Biosciences	B6NTac
Mouse: (Pw11, RIAD)		NA
Mouse (Pw9, CTR)		NA
Mouse: Ldlr^-/-^ (C57BL6/J)	Jackson Laboratory	#002207
Mouse: Ob/Ob (C57BL6/J)	Jackson Laboratory	#000632
Mouse: Rag2-/-	Taconic Biosciences	Model RAGN12

**Software and algorithms**		

FlowJo	BD	10.7.1.
Helios		
Cytobank		
Illumina`s bcl2fastq		v2
Seurat		V 4.0.1
STACASR package		
Fastp software		v0.20.1
Salmon		v1.5.2
DESeq2		v1.32.0
tximeta		v.1.12.0
NicheNetr R package		
GraphPad Prism		9

**Other**		

Metabolic home cages	Promethion System; Sable Systems, Las Vegas, Nevada, USA	NA
EchoMRI	EchoMRI 100-Analyzer; EchoMRI, Houston, Texas, USA	NA
High fat diet containing 45 kcal% fat	Research Diets, Inc	Diet# D16031603
High fat diet containing 60 kcal% fat	Altromin	Diet # c1090-60
Parr 6100 compensated calorimeter	Parr Instrument Company, Moline, IL	NA
Accu-Chek Aviva	Roche	886877
Corning cell strainer, 100 μm	Merck	CLS431752-50EA
BD FACS Verse Flow cytometer	BD	NA
autoMACS pro separator	Miltenyi Biotec	NA
Helios CyTOF Mass Cytometer	Standard BioTools	NA
NextSeq500	Illumina	NA

### Resource availability

#### Lead contact

Further information and requests for resources and reagents should be directed to and will be fulfilled by the lead contact, Bente Halvorsen (bente.halvorsen@medisin.uio.no).

#### Materials availability

This study did not generate new unique reagents.

#### Data and code availability

• Single-cell RNA-seq data and bulk RNA seq data have been deposited at NCBI Sequence Read Archive and are publicly available as of the date of publication. Accession numbers are listed in the key resources table.
• No original code was developed for this project. All bioinformatic analyses were based on established pipelines, as documented via their respective methods and references.
• Any additional information needed to reanalyze the data reported in this paper is available from the lead contact upon request.

See key resources table for details.

### Experimental model and study participant details

#### Ethical statement for animal experiments

This study has been approved by the Institute for Laboratory Animal Research Guide for the Care and Use of Laboratory Animals and was approved by the National Committee for Animal experiments, the Netherlands, or the Norwegian National Animal Research Authority with project license numbers FOTS 19685, 21003 and 23000. All animal experiments were performed in accordance with the European Directive 2010/63/EU and conducted in accordance with Animals in Research: Reporting *in vivo* Experiments (ARRIVE) guidelines.

#### Mouse models

The RIAD transgenic mice were generated on a C57BL/6 background (Taconic Biosciences), as previously described.^7^ Briefly, the mice express a HA-tagged 100 amino acid protein with the high-affinity RI anchoring disruptor (RIAD) in the context of the helical part of the AKAP ezrin under the control of the Lck distal promoter, which causes disruption of PKA I signaling in cells where this promoter is active, namely T cells and NK cells.^7^ Control mice were wild type (WT) siblings or a strain expressing a disrupted RIAD construct (Pw9p). The mice were born and housed at three different locations; the Department for Comparative Medicine, Oslo University Hospital Rikshospitalet, Oslo, Norway; Section of Comparative Medicine, Institute of Basic Medical Sciences, University of Oslo, Norway and Einthoven Laboratory for Experimental Vascular Medicine, Leiden University Medical Center, Leiden, The Netherlands. The founders of the *Rag2*^*-/-*^ mice used in this study were purchased from Taconic Biosciences and further bred and housed at the Department for Comparative Medicine, Oslo University Hospital Rikshospitalet, Oslo, Norway. *Ldlr*^*-/-*^ and *Ob/Ob* mice were obtained from Jackson Laboratory and acclimatized for one week before entering the experiment. The sex and age of the mice used in the different experiments has been stated in the result sections and Figure legends, respectively.

All mice were housed under a 12-hour light–dark cycle with *ad libitum* access to food and water unless otherwise stated. At the end of the experiments, the mice were sacrificed by cardiac puncture. Blood was collected using a 1 mL syringe with coating of 0.5 M EDTA (VWR Chemicals). EDTA blood was immediately placed on ice and centrifuged at 2000g (4°C) for 20 min to obtain platelet-poor plasma. Organs were removed immediately and weighted. All samples were stored at −80°C or on formalin until further use.

#### Group size considerations

The number of animals included in the individual experiments is based on prior experience with similar studies with this genetic model. Group sizes reflect our ambition to maintain animal welfare and standardize all experimental conditions, i.e. by providing similar environmental stimuli and avoiding solitary housing. Additionally, the group sizes allow us the flexibility to remove mice reaching humane end-points without compromising the statistical validity of the experiment.

### Method details

#### Metabolic mouse study

Female mice (5 RIAD + 5 control mice) and male mice (8 RIAD + 8 control mice) were acclimatized for at least 24 hours before being housed individually in automated metabolic home cages (Promethion System; Sable Systems, Las Vegas, Nevada, USA) for 5 days. In these cages, data on voluntary locomotor behavior (by beam breaks), food intake, O2 consumption, and CO2 production were continuously collected in 5-min bins and energy expenditure and respiratory exchange ratio (RER) were calculated. Body composition was determined by EchoMRI (EchoMRI 100-Analyzer; EchoMRI, Houston, Texas, USA). Blood was collected in paraoxon-coated capillaries from 4 hour fasted mice to isolate plasma and measure triglycerides and cholesterol using enzymatic kits (Roche Diagnostics, Mannheim, Germany) and free FA (NEFA-C kit, Wako Diagnostics, Instruchemie, Delfzijl, the Netherlands).

#### *Ex vivo* lipolysis assay

Viceral, epidydymal adipose depots were dissected from 3 month old RIAD mice (n=5) and control mice (n=5) to assess *ex vivo* lipolysis of visceral adipose tissue, as previously described by Roy et al.,^25^ with minor adjustments. Briefly, ∼50 mg of adipose depots were incubated in KRBH buffer with or without isoproterenol for 2 hours. Media was harvested and frozen at -80°C. Glycerol and NEFA were measured in the media with the use of Glycerol Assay kit (Cell based, ab133130; Abcam) and Free Fatty Acid Fluorometric Assay Kit (Item no. 700310; Cayman Chemicals), and normalized to individual fat pad weight.

#### Measurement of fecal calory loss

RIAD (n=7) mice and control mice (n=8) at 4-5 months of age were fed a HFD (Diet# D16031603, containing 45 kcal% fat, Research Diets, Inc.), for 1 week before they were moved into separate cages and feces was collected over 48 hours. Fecal calories were determined essentially as described.^26^ Briefly, ∼300 mg dried feces was ground and combusted in a Parr 6100 compensated calorimeter (Parr Instrument Company, Moline, IL) using a 1108 Oxygen Bomb placed in 2000 g of demineralized water. The caloric content of the samples was derived from the temperature increase of the water, using benzoic acid as a standard.

#### Oral glucose tolerance test

Male (2.5 months old) and female (4 months old) RIAD mice (n=10/8) and control mice (n=6/8) were fasted for 4 hours prior to oral glucose tolerance test (OGTT). Glucose dissolved in water was given at a dose of 2 g/kg body weight, and circulating blood glucose was measured from the tail vein using Accu-Chek Aviva (Roche) for 120 minutes after gavage.

#### Cell isolation and single-cell suspension from adipose tissue

Visceral white adipose tissue from RIAD mice (n=3) and WT mice (n=3) were excised and cut into small pieces on ice before being incubated in Hanks’ Balanced Salt Solution (Sigma) containing 1 mg/mL Type II Collagenase (Sigma), during vigorously shaking at 37°C for 30 min. EDTA was added to a final concentration of 10 mM before 7 min additional incubation at 37°C. The single-cell suspension was passed through a 100 μm nylon filter, washed with 10 mL FACS buffer, and centrifuged at 500g for 10 min at 4°C for collection.

#### HA-tag quantification by flow cytometry

For HA-tag quantification, spleen, thymus, visceral WAT, subcutaneous WAT and BAT was harvested from euthanized animals. Blood was collected from the calf vein. Adipose tissue were minced in small pieces with scissors and subsequently digested with type II collagenase (1 mg/mL in 10 mL per g) for 30 min at 37°C with vigorous shaking. EDTA was then added to a final concentration of 10 mM and incubated for 7 min at 37°C. For a single cell suspension, digested adipose tissue, and undigested spleen and thymus, respectively were pressed through a 70 μm nylon filter and centrifuged at 300 g for 10 min. To remove red blood cells from the adipose tissue, spleen, thymus and the collected blood, the samples were incubated for 5 min on ice with lysis buffer (Cat # 420302, BioLegend), washed in flow buffer (PBS, 2 mM EDTA, 0.5% BSA) and pelleted at 300g for 10 min. Cells were resuspended in anti-CD3 (cat. no. 130-117-671, Macs Miltenyi Biotec) for all samples; for tissues also anti-CD4 (cat. no. 560181, BD Biosciences) and anti-CD8 (cat. no. 563068, BD Biosciences), and incubated in the dark for 15 min at room temperature. After wash and centrifugation (x2) in flow buffer, the cells were fixed by adding 100 μL fix solution (Fixation/Permeabilization kit, cat. no. 554714, BD Biosciences) for 1 hour at 4°C. The cells were further washed in permeabilization solution (Fixation/Permeabilization kit), pelleted at 500g for 10 min and resuspended in anti-HA (Macs, cat. no. 120-002-687) for 30 min of incubation in the dark at 4°C. Finally, the cells were washed in permeabilization buffer, resuspended in flow buffer and run on a flow cytometer (BD FACS Verse) and analyzed using FlowJo 10.7.1.

#### Bone marrow chimeras

C57BL/6NTac (n=5-8), *Ldlr*^*-/-*^ (C57BL6/J, n=12-14) and *Ob/Ob* (C57BL6/J n=6-7) mice were irradiated with 6 Gray × 2 with 4 hours between each radiation cycle. The following day, donor bone marrow was isolated from the femurs and tibias of RIAD mice. Control bone marrow was isolated from WT siblings or a strain expressing a disrupted RIAD construct (Pw9p). Briefly, the bones were rinsed with 70% ethanol and ice-cold PBS under sterile conditions. The epiphyses of each bone were cut, and bone marrow cells were flushed with 10 mL ice-cold PBS and filtered through a 40 μm nylon filter. The irradiated C57BL/6NTac, *Ldlr*^*-/-*^ and *Ob/Ob* mice received 1.0 × 10^6^ bone marrow cells (in 100 μL PBS) from the donor mice through tail vein injections, and acidic (pH 2.5) sterile water was provided *ad libitum* until termination. Bone marrow from WT siblings was used as a control in C57BL/6NTac and *Ob/Ob* recipient mice, while bone marrow from Pw9p was used as a control in *Ldlr*^*-/-*^ recipient mice. Blood samples were taken from the calf vein after 8 weeks to verify engraftment through detection of the HA-tag with flow cytometry. Once adequate engraftment was verified, C57BL/6NTac and *Ldlr*^*-/-*^ recipient mice were fed a HFD (Diet # c1090-60, containing 60 kcal% fat, Altromin and Diet# D16031603, containing 45 kcal% fat, Research Diets, Inc., respectively), to accelerate obesity development, while *Ob/Ob* recipient mice were fed standard rodent chow until termination. Mice that spontaneously died before the end of the experiment were excluded from further analysis.

#### Adoptive transfer of control or RIAD T cells into *Rag2*^*-/-*^ mice

Spleens were harvested from control (C57BL/6Ntac) and RIAD mice (1.5-2 months of age), minced and processed to obtain a single-cell suspension. Red blood cells were lysed with RBC lysis buffer (Cat # 420302, BioLegend), following the manufacturer’s instructions. T cells were isolated from the singe-cell suspension using the pan T cell isolation kit II (Cat # 130-095-130, Milteny Biotec) and the autoMACS pro separator. Isolated T cells (1.0 × 10^7^) from control (n=6) or RIAD (n=5) mice were transferred to *Rag2*^*-/-*^ (Taconic, 1.5-2 months of age) mice through tail-vein injection (100 μL in PBS). Recipient mice were randomized prior to T cell transfer. To accelerate obesity the mice were then fed a HFD and the weight of the recipient mice was monitored regularly. The presence of WT and RIAD T cells in blood were analyzed by flow cytometry after 14 weeks and at harvest, 24 weeks after the T cell transfer. At harvest, also the presence of T cells in tissues were determined.

#### Mass cytometry antibody conjugation

Three million cells from bone marrow, thymus, blood or WAT were stained for mass cytometry with metal-conjugated monoclonal antibodies as listed in Table S3. Briefly, cells were stained with 5 μM cisplatin in PBS without calcium or magnesium for 5 min at room temperature and quenched by Maxpar staining buffer (Fluidigm). All cells to be analyzed were barcoded using the Cell-ID 20-Plex Pd Barcoding kit from Fluidigm, and pooled before incubation with Fc block for 10 min at room temperature. Surface epitopes were recognized using the antibody cocktail listed in Table S3 for 20 min in Maxpar staining buffer. Antibodies were either directly conjugated antibodies purchased from Fluidigm or alternatively purchased in a carrier-protein-free format and conjugated with the respective metal Maxpar X8 Multi-Metal labeling kits (Fluidigm). Following wash with Maxpar staining buffer, cells were fixed in 1.6% paraformaldehyde for 5 min at room temperature before permeabilization by resuspension in 1 mL ice-cold methanol. Intracellular epitopes were recognized using the antibody cocktail listed in Table S1 for 20 min in Maxpar staining buffer. For cell identification, cells were washed in staining buffer and stained with DNA intercalator (Fluidigm) containing natural abundance Iridium (191Ir and 193Ir) prepared to a final concentration of 125 nM. Prior to analysis, the cells were washed in cell acquisition buffer (CAS; Fluidigm) before resuspension in CAS with a 1:10 dilution of EQ four element calibration beads (Fluidigm) and filtered through a 35 μm nylon mesh filter cap. Samples were acquired on a Helios CyTOF Mass Cytometer (Fluidigm) at an event rate of 500 events per second or less. Data was normalized (including EQ bead-removal) and debarcoded using Helios software.

#### Single-cell sequencing of peripheral blood mononuclear cells (PBMCs)

PBMCs for single-cell sequencing were isolated from a single RIAD and WT mouse according to 10x Genomics® Single Cell Protocols Cell Preparation guide. Cells were resuspended in PBS +0.04% BSA and immediately processed at the Genomics Core Facility (Oslo University Hospital) using the Chromium Single Cell 3/ Library & Gel Bead Kit v2 (Chromium Controller System, 10X Genomics). The sequencing libraries were generated following the recommended protocol. Sequencing was performed on a NextSeq500 (Illumina) with 5∼% PhiX as spike-inn. Sequencing raw data were converted into fastq files by running the Illumina`s bcl2fastq v2.

#### RNA sequencing of adipose tissue

White adipose depots were dissected from the transplanted *Ob/Ob* mice and kept on ice-cold PBS (< 1 hour) before the experimental procedure (from n=3 mice receiving control bone marrow + 3 mice receiving RIAD bone marrow). Explants were cut into macroscopically similar pieces (2x2x2 mm) and incubated in DMEM-F12 (Gibco, Fisher Scientific, UK) with 1% penicillin/streptomycin (Gibco) and with 1% HSA (endotoxin-free; Sigma Aldrich, St. Louis, MI, USA). After 18 hours the tissue samples and conditioned media were harvested and stored (-80°C) until RNA extraction and further analyses.

RNA from white adipose tissue was isolated with a RNeasy Mini kit (Qiagen, Hilden, Germany) according to manufacturer’s protocol, and sent to Novogene (UK) Company Limited for stranded library preparation and sequenced in 150bp pair-end mode. Contaminated adapters and low-quality reads with phred score below 30 in the raw sequencing files were filtered out by the fastp software (v0.20.1).^27^

#### Immunofluorescence detection of HA-tag

Adipose tissues were formalin-fixed, dehydrated, embedded in paraffin, and sliced into 5-μm-thick cross-sections using a rotary microtome. The presence of HA-tag expression was detected using anti-HA-Tag (rabbit mAb, 1:200, Cell Signalling) and secondary antibody (goat-anti-rabbit IgG, Alexa Fluor 488, Thermo Fisher). Stained sections were sealed using ProLong™ Gold Antifade Mountant with DNA Stain DAPI (Invitrogen).

### Quantification and statistical analysis

#### Analysis of CyTOF data

FCS-files were uploaded to cytobank and manual gating was performed to gate out EQ beads, aggregated events (Ir191 and Ir193) and dead cells (cisplatin) before identifying main leukocyte populations (CD45^+^, B cells, T cells, macrophages, dendritic cells etc.). The gating scheme is outlined in Figure S5. We performed viSNE analysis, which is a dimensionality reduction technique for high-dimensional single-cell data.^28^ The CD45^+^ population was selected with equal down sampling based on the lowest event count in all samples. The following parameters were used: iterations: “4000”, perplexity: “50”, and theta: “0.5” (final divergence at 3.191771), all channels were used for dimensionality reductions except CD45 and HA-tag.

FlowSOM clustering was performed, which partitions all individual cells based on their marker expression into clusters and metaclusters using Self-Organizing Maps (SOM) and is visualized in a Minimum Spanning Tree (MST).^29^ FlowSOM was performed with the following parameters: Event sampling method: “CD45+ events”; Clustering Method: “Hierarchial Consensus”; Number of metaclusters: “10”; Number of clusters: “100”; Iterations: “100”; all channels were used for clustering except CD45 and HA-tag.

#### Quantification and analysis of RNA sequencing data

To analyze the single-cell sequencing data, the processed 10x files were analyzed in R using Seurat version 4.0.1.^30^ Quality control was performed to remove doublets based on the number of counts, the number of features and the percentage of mitochondrial genes in the cells. 7464 cells from the WT and 9262 cell from the RIAD mouse passed the control. Samples were integrated by STACASR package.^31^ The clustering of cells was based on the gene expression count matrix for each cell. A PCA was first performed to determine the principal components (PCs) for the integrated data set. These are further used to calculate the distance matrices for the nearest neighbor clustering. The number of PCs that describe most of the data set was first identified based on an elbow plot or a jackstraw plot. For this data set, the number of dimensions (dims) for the FindNeighbors function was set to 1:20, visualized as UMAP. The FindAllMarkers function within seurat was invoked to determine the genes which were differentially expressed between the seurat clusters for the integrated data sets. The parameter min.pct was modified to 0.25 from the default 0.1 to ensure that the genes labeled as markers are present in at least 25% of either of the two populations (clusters) being tested. For re-clustering, the CD3 positive clusters (0, 2, 3, 4, 6, 8, 11 and 12) were selected. The raw gene counts of cells involved in these clusters were re-scaled and the same clustering method and parameters described above were applied again.

To map and quantify gene expression from RNA sequencing of adipose tissue, Salmon (v1.5.2) was used to map the filtered reads to the mouse transcriptome (Gencode Mouse Release M27) with 200 bootstrap iterations for quantification.^32^^,^^33^ To obtain the differentially expressed genes (DEGs), the Salmon outputs were imported into DESeq2 (v1.32.0) via tximeta (v.1.12.0).^34^^,^^35^ Gene with p value below 0.05 were considered significant DEGs and uploaded to Metascape for gene functional enrichment analysis.^36^

#### Prediction of T cell expressed ligands and adipose tissue receptors interactions

We used the NicheNetr R package to predict the T cell expressed ligands and adipose tissue receptors interactions that might drive gene expression changes in the latter.^11^ In this analysis, NicheNet’s ligand-target prior model is used. Ligand genes detected in the RNA-seq of RIAD T cells are considered potential ligands. Receptors genes detected in the RNA-seq of the visceral adipose tissue are considered potential receptors. DEGs involved in specific GO terms were defined as genes of interest and all genes expressed in visceral adipose tissue are set as background. Then NicheNet algorithm were performed to test the regulatory potential between the ligands and DEGs involved in specific GO terms. Isolation of spleen T cell RNA and RNA seq was performed as described above from RIAD and control mice, with the only exceptions being the sequencing center (Norwegian Sequencing Center), and the number of cycles used during sequencing (151).

#### Statistical analysis

The software package GraphPad Prism 9 was used for statistical analyses. Differences between groups was assessed by T test or Mann Whitney test. AUC curves were compared as described by Graphpad Prism. Significance was considered at p<0.05.

## Supplemental information

Document S1. Figures S1–S10 and Tables S1–S3

File S1. Markers for clusters identified in PBMC scRNA seq

File S2. Markers for T cell clusters identified in scRNA seq of PBMC

File S3. Differently expressed genes separating T cell cluster 1, 2 and 4 in scRNA seq. of PBMC

File S4. Differently expressed genes from RNA seq. of white adipose tissue

File S5. Potential ligands identified in T cell RNA seq by NicheNetr R package
