# Supplementary material for: T cells with increased responsiveness cause obesity in mice without diet intervention
Source: iScience. 2024 Mar 11;27(4):109471. doi: 10.1016/j.isci.2024.109471 (PMC10973599; doi:10.1016/j.isci.2024.109471)
Supplement: Document S1. Figures S1–S10 and Tables S1–S3 [file mmc1.pdf]

iScience, Volume *27*

Supplemental information

T cells with increased responsiveness

cause obesity in mice without diet intervention

Ida Gregersen, Xiang Y. Kong, Sander Kooijman, Håvard Foyn, Helene Grannes, Maria B. Olsen, Anna M. Lone, Kuan Yang, Ana Quiles-Jiménez, Marianne Tran, Jonas Øgaard, Filip M. Segers, Azita Rashidi, Ellen Lund Sagen, Knut H. Lauritzen, Amanda C.M. Pronk, Jan Freark de Boer, Kirsten B. Holven, Espen Melum, Pål Aukrust, Kjetil Taskén, Sverre Holm, Patrick C.N. Rensen, Tuva B. Dahl, and Bente Halvorsen

Supplementary tables

Table S1. Organ weights from Male and Female mice, related to Figure 1

Organ weights from Male (right) and Female (left) Wt (CTR) and Transgenic (RIAD) mice fed chow diet. gWAT (gonadal white adipose tissue), sWAT (subcutaneous white adipose tissue), iBAT (intrascapular brown adipose tissue), sBAT (subscapular brown adipose tissue), TA (tibialis anterior muscle), EDL (extensor digitorum longus muscle), Sol (soleus muscle).

Organ	CTR (n=8)	RIAD (n=7)	p-value	CTR (n=5)	RIAD (n=5)	p-value
Liver	1,287 ± 0,168	1,836 ± 0,574	0.021	0,999 ± 0,172	0,822 ± 0,272	0.222
gWAT	0,454 ± 0,299	1,335 ± 0,174	0.0003	0,357 ± 0,161	0,851 ± 0,428	0.032
sWAT	0,339 ± 0,149	1,550 ± 0,228	0.0003	0,202 ± 0,082	0,405 ± 0,205	0.095
iBAT	0,117 ± 0,042	0,228 ± 0,075	0.006	0,040 ± 0,012	0,103 ± 0,080	0.143
sBAT	0,114 ± 0,029	0,158 ± 0,044	0.004	0,008 ± 0,003	0,034 ± 0,012	0.016
TA	0,104 ± 0,005	0,073 ± 0,011	0.0003	0,042 ± 0,007	0,029 ± 0,007	0.056
EDL	0,023 ± 0,003	0,017 ± 0,004	0.004	0,008 ± 0,003	0,008 ± 0,002	0.889
Sol	0,025 ± 0,002	0,020 ± 0,003	0.005	0,010 ± 0,001	0,008 ± 0,002	0.151

Data analyzed with Mann Whitney and presented as mean±SD.

Table S2. Differential count of blood cells in CTR and RIAD mice, related to Figure 1.

Differential count of blood cells in CTR (n=6) and RIAD (n=6) mice. WBC (White blood cells; $10^3/\mu L$). RBC (Red blood cells; $10^6/\mu L$). HGB (Hemoglobin; g/dl). HCT (Hematocrit; %). MCV (mean corpsular volume; μm^3). MCH (Mean corpsular hemoglobin; pg). MCHC (Mean corpsular hemoglobin concentration; g/dl). RDW (Red cell distribution with; %). PLT (platelets; $10^3/\mu L$). MPV (Mean platelet volume; μm^3). PDW (Platelet distribution with. %). %LYM (Lymphocytes). %MON (Monocytes). %GRA (Granulocytes). #LYM (Lymphocytes; $10^3/\mu L$). #MON (Monocytes; $10^3/\mu L$). #GRA (Granulocytes; $10^3/\mu L$).

	**CTR**	**RIAD**	**p-value**
**WBC**	9.2 (8.9-12.3)	11.1 (8.3-14.2)	0.563
**RBCD**	8.3 (8.0-8.6)	7.8 (7.5-8.4)	*0.048*
**HGB**	15.9 (15.0-16.4)	16.2 (15.7-.18.0)	0.290
**HCT**	45.1 (44.0-46.5)	44.2 (43.2-46.2)	0.383
**MCV**	55.0 (54.0-55.0)	57.0 (55.0-58.0)	*0.01*
**MCH**	19.00 (18.7-19.7)	20.3 (20.7-21.5)	*0.002*
**MCHC**	35.1(34.1-36.0)	36.8 (35.5-37.4)	*0.004*
**PLT**	645.5 (553.0-747.0)	625.5 (594.0-849.0)	0.900
**MPV**	7.9 (5.1-8.7)	7.1 (6.2-7.6)	0.368
**RDW**	13.5 (13.5-13.9)	13.4 (13.4-13.9)	0.435
**%LYM**	91.5 (67.1-92.4)	89.2 (87.9-93.6)	0.818
**%MON**	6.3 (5.7-25.5)	8.0 (5.2-9.2)	0.818
**%GRA**	2.5 (1.9-7.4)	2.7 (1.2-4.8)	0.619
**#LYM**	8.4 (5.9-11.7)	10.0 (7.6-13.2)	0.393
**#MON**	0.7 (0.5-2.2)	0.7 (0.6-1.1)	0.900
**#GRA**	0.4 (0.3-0.8)	0.3 (0.3-0.6)	0.621

Presented as median and min-max. Analyzed with Mann Whitney.

Table S3. List of metal conjugated monoclonal antibodies used for mass cytometry, related to Figure 3.

Metal	Target	Clone	Reactivity	Source	Catalogue Number	Dilution
141Pr	Ly-6G	1A8	Mouse	Fluidigm	3141008B-DVS	1/100
142Nd	CD185	L138D7	Mouse	Fluidigm	3142015B-DVS	1/100
143Nd	TCRβ	H57-597	Mouse	Fluidigm	3143010B-DVS	1/100
145Nd	CD69	H1.2F3	Mouse	Fluidigm	3145005B-DVS	1/100
146Nd	F4/80	BM8	Mouse	Fluidigm	3146008B-DVS	1/100
147Sm	CD45	30-F11	Mouse	Fluidigm	3147003B-DVS	1/100
148Nd	CD11b	M1/70	Mouse	Fluidigm	3148003B-DVS	1/100
149Sm	CD19	6D5	Mouse	Fluidigm	3149002B-DVS	1/75
150Gd	CD80	16-10A1	Mouse	Biolegends	104735	1/100
151Eu	CD25	3C7	Mouse	Fluidigm	3151007B-DVS	1/100
152Sm	CD3ε	145-2C11	Mouse	Fluidigm	3152004B-DVS	1/100
153Eu	HA-Tag	EPR22819-101	Species independant	Abcam	Ab256483	1/100
154Sm	CTLA-4	UC10-4B9	Mouse	Fluidigm	3154008B-DVS	1/100
155Gd	iNOS	CXNFT	Mouse	Fisher Scientific	15567456	1/100
156Gd	CD1d	1B1	Mouse	Biolegends	123502	1/100
158Gd	FoxP3	FJK-16s	Mouse	Fluidigm	3158003A-DVS	1/100
159Tb	CD279 (PD-1)	J43	Mouse	Fluidigm	3159023B-DVS	1/100
160Gd	CD62L	MEL-14	Mouse	Fluidigm	3160008B-DVS	1/100
161Dy	Ki-67	B56	Human/Mouse/Rat	Fluidigm	3161007B-DVS	1/100
162Dy	Tim-3	RMT3-23	Mouse	Fluidigm	3162029B-DVS	1/100
165Ho	CD161 (NK1.1)	PK136	Mouse	Fluidigm	3165018B-DVS	1/100

167Er	CD335 (Nkp46)	29A1.4	Mouse	Fluidigm	3167008B-DVS	1/100
168Er	CD8α	53-6.7	Mouse	Fluidigm	3168003B-DVS	1/100
169Tm	CD206	C068C2	Mouse	Fluidigm	3169021B-DVS	1/100
170Er	CD169	3D6.112	Mouse	Fluidigm	3170018B-DVS	1/100
171Yb	CD44	IM7	Human/Mouse	Fluidigm	3171003B-DVS	1/400
172Yb	CD4	RM4-5	Mouse	Fluidigm	3172003B-DVS	1/100
174Yb	I-A/I-E (MHC II)	M5/114.15.2	Mouse	Fluidigm	3174003B-DVS	1/100
175Lu	CD127	A7R34	Mouse	Fluidigm	3175006B-DVS	1/100
176Yb	ICOS	7E.17G9	Mouse	Fluidigm	3176014B-DVS	1/100

Supplemental Figures

a

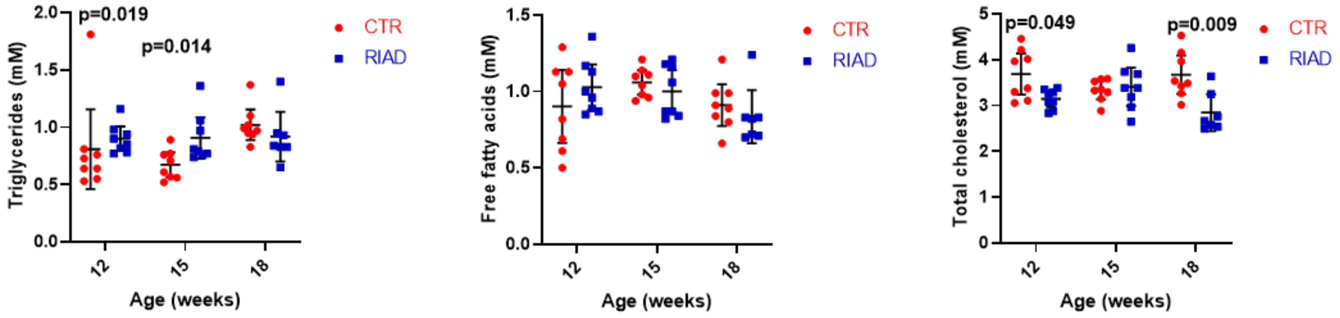

b

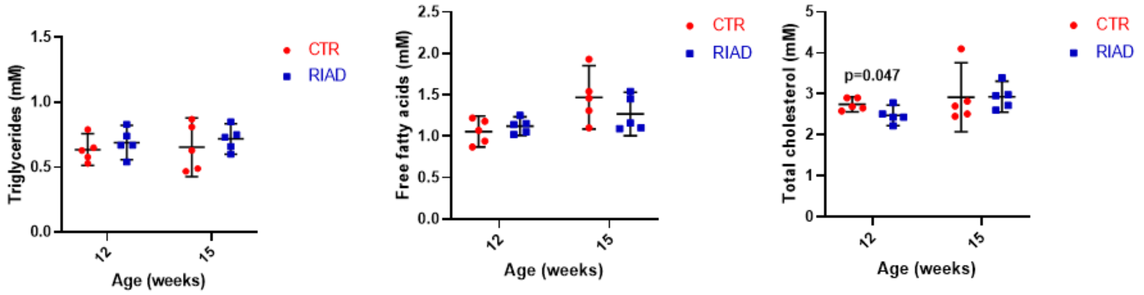

Figure S1. Plasma lipid levels of male and female mice, related to Figure 1.

a) Plasma levels of triglycerides, free fatty acids, and total cholesterol in male mice at 12, 15 and 18 weeks of age, CTR (n=8) and RIAD (n=8), fed chow diet. **b)** Plasma levels of triglycerides, free fatty acids, and total cholesterol in female mice at 12 and 15 weeks of age, CTR (n=5) ang RIAD (n=5), fed chow diet, data presented as mean and 95% CI and analyzed with Mann Whitney test

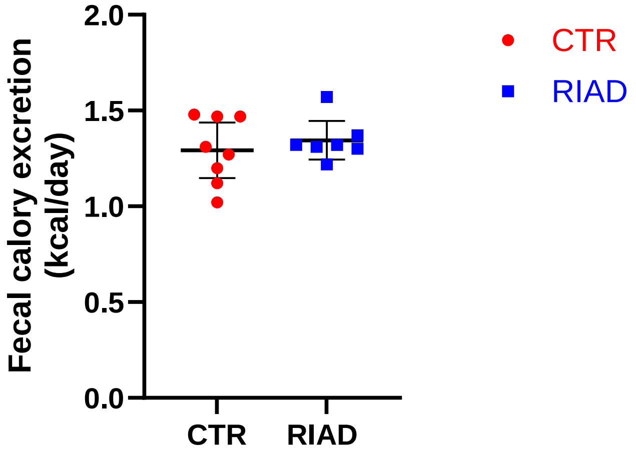

Figure S2. Fecal energy excretion from RIAD and control mice, related to Figure 1.

Fecal energy excretion measured in feces collected from female control (CTR, n=8) and transgenic mice (RIAD, n=7) at 4-5 months of age. 48 hour fecal calorie excretion presented as kcals per day. Data analyzed with Mann Whitney test and presented as mean and 95% C.

a

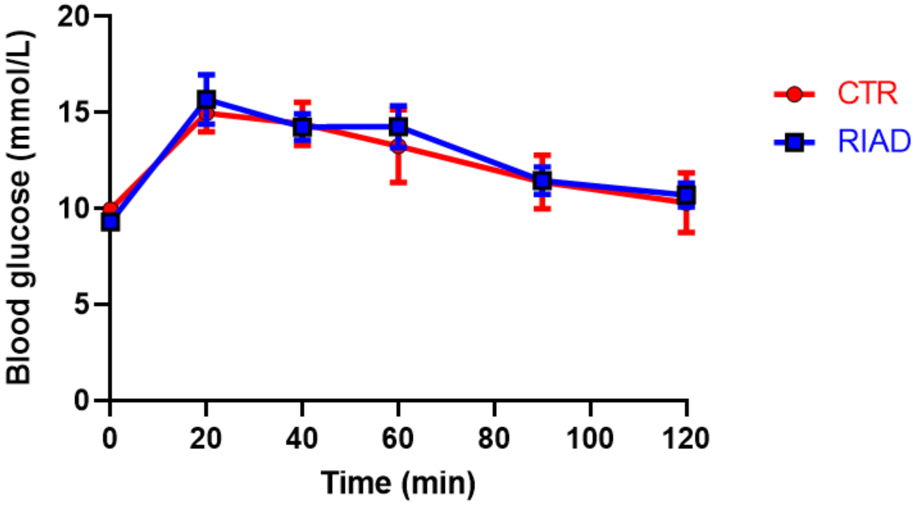

Male

b

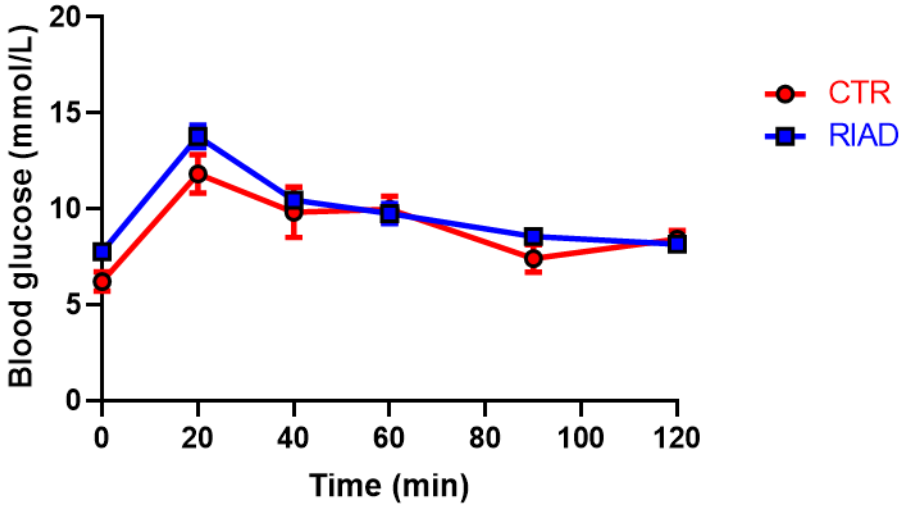

Female

Figure S3. Oral glucose tolerance test of male and female mice, related to Figure 1.

Blood glucose measured before and five times after an oral glucose challenge in **a)** Male (2.5 months old) and **b)** female (4 months old) RIAD (n=10/8) and CTR mice (=6/8).

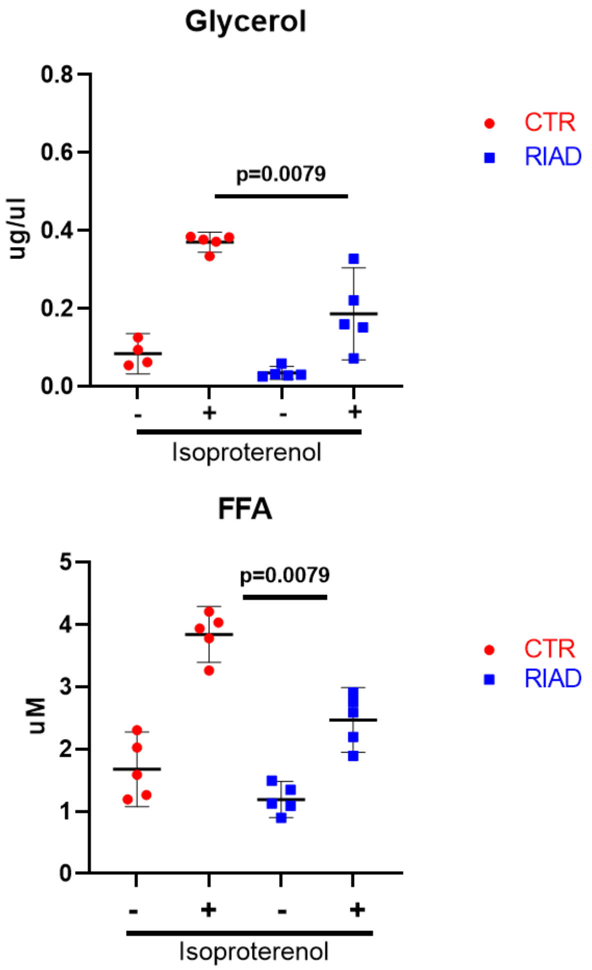

Figure S4. Ex vivo lipolysis assay, related to Figure 1.

Release of glycerol (top) and non-esterified free fatty acids (NEFA, bottom), from viceral adipose tissue explants stimulated with or witout isoproterenol. Three months old RIAD mice (n=5) and control mice (n=5). Data analyzed with Mann Whitney test.

a

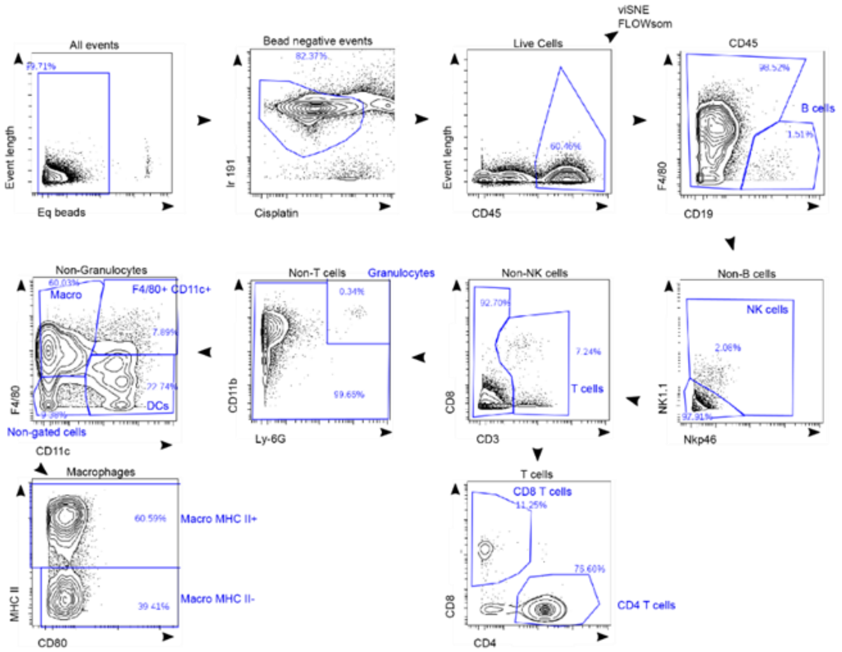
All events
Event length
Eq beads
Bead negative events
Ir 191
Cisplatin
Live Cells
CD45
viSNE
FLOWsom
CD45
F4/80
CD19
B cells
Non-B cells
NK cells
NK1.1
Nkp46
Non-NK cells
T cells
CD8
CD3
Non-T cells
Granulocytes
CD11b
Ly-6G
Non-Granulocytes
Macro
F4/80+ CD11c+
DCs
Non-gated cells
CD11c
Macrophages
Macro MHC II+
Macro MHC II-
MHC II
CD80
T cells
CD8 T cells
CD4 T cells
CD4

b

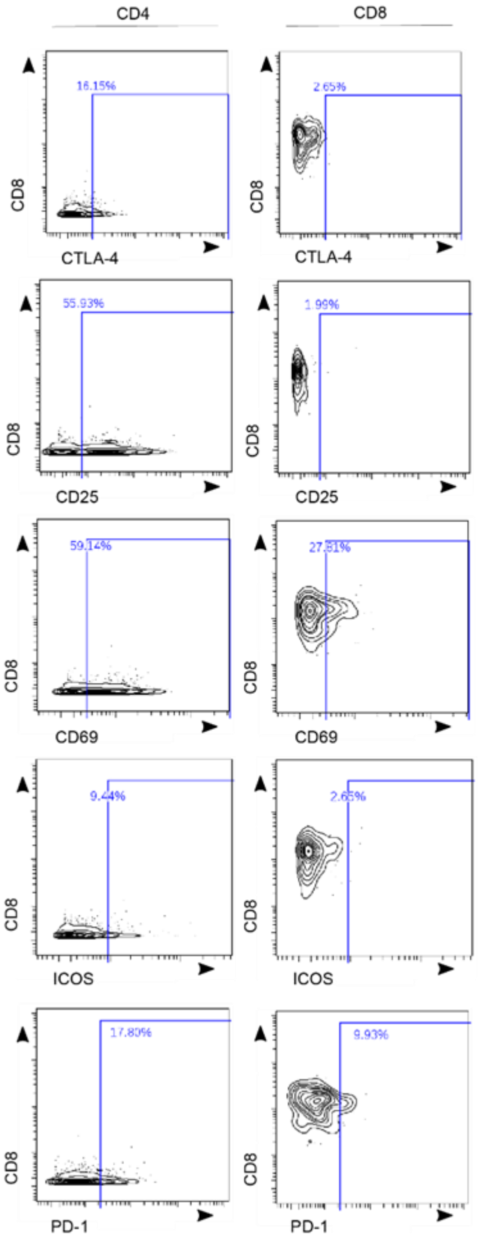
CD4
CD8
CTLA-4
CD25
CD69
ICOS
PD-1
16.15%
2.65%
55.93%
1.99%
59.14%
27.81%
9.44%
2.65%
17.80%
9.93%

Figure S5. Gating hierarchy for the manually gated cell populations, related to Figure 3.

a) Overall gating hierarchy for the manually gated cell populations. First, all events positive for EQ beads were gated out before single cells were gated using Cisplating and Ir191. Among these, The $CD45^+$ population was gated and this population was used for generating viSNE plots and FLOWsom clustering. B cells were gated using CD19, NK cells were identified using Nkp46 and NK1.1, T cells were identified using CD3, CD4 and CD8, while granulocytes were gated using CD11b and Ly-6G. Dendritic cells were identified as cells positive for CD11c while negative for F4/80. FLOWsom clustering identified a potentially differentiated population double-positive for F4/80 and CD11c. Macrophages were first identified as F4/80+ CD11c- cells before these were split based on the presence of MHC II. **b)** Gates used within the $CD4^+$ and $CD8^+$ cells to identify CTLA-4^+, $CD25^+$, $CD69^+$, $ICOS^+$ or PD-1^+ T cells.

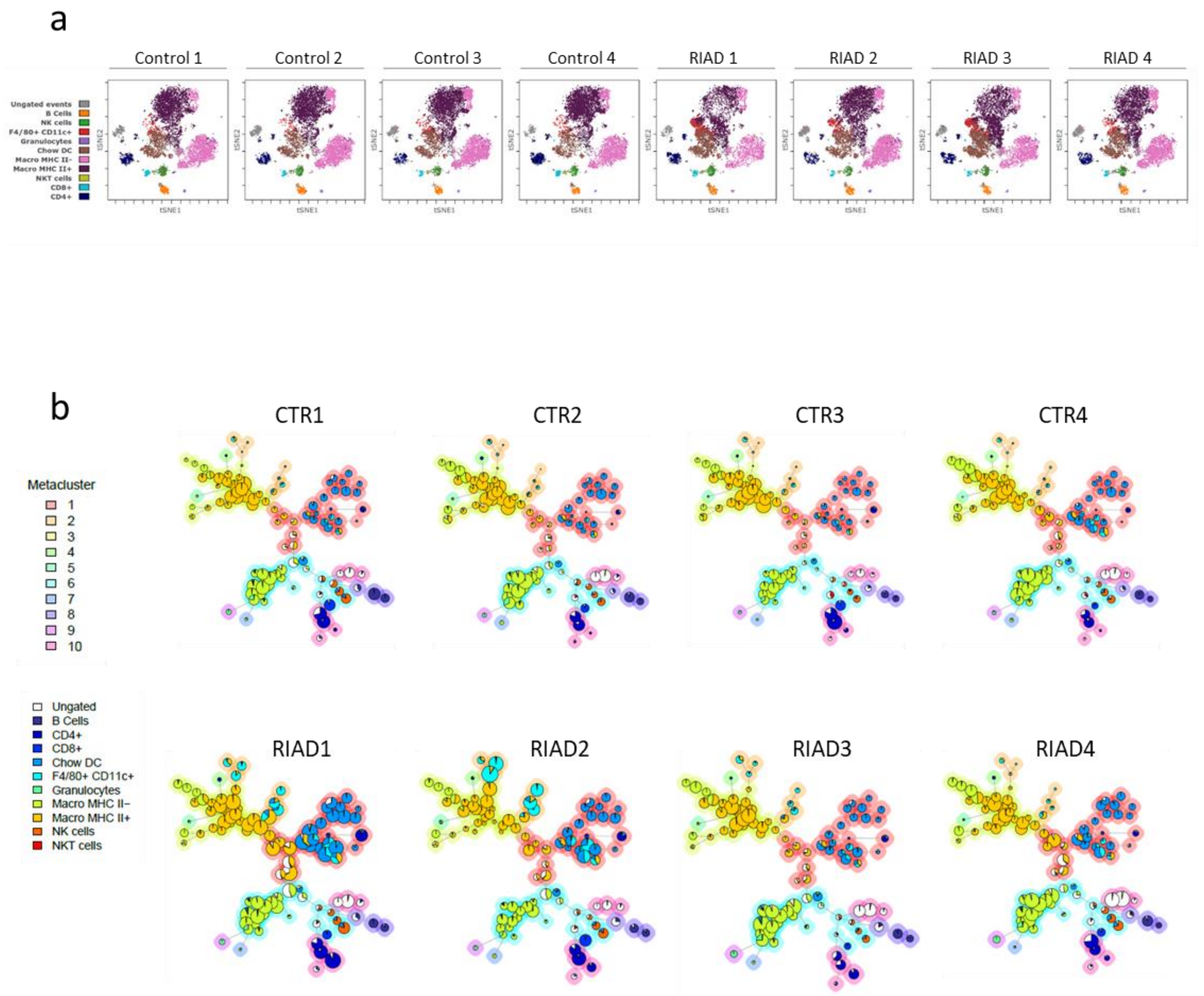

Figure S6. Mass cytometry analysis (CyTOF) of visceral WAT from RIAD and control mice, related to Figure 3.

a) viSNE plots of CD45+ events of cells harvested from Control and RIAD WAT (4+4). Equal number of events in each plot. Plots are overlaid with colors representing the type of cells identified through manual gating. DCs are defined as CD11c positive cells and macrophages as F4/80 positive cells. Macrophages were split based on their expression of MHC II. In addition, a F4/80^{+} CD11c^{+} population was identified that were mainly present in the RIAD. **b)** FlowSOM clustering. All replicates for supplemental. Color of Piechart indicate manually gated cell populations.

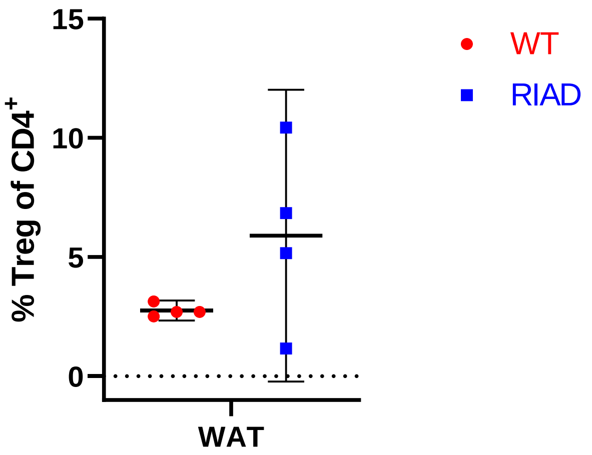

Figure S7. T regulatory cells in RIAD and WT adipose tissue, related to Figure 3.

Percentage T regulatory (Treg) cells of CD4$^+$ cells in RIAD and WT white adipose tissue (WAT).

n=4+4, data analyzed with Mann Whitney test

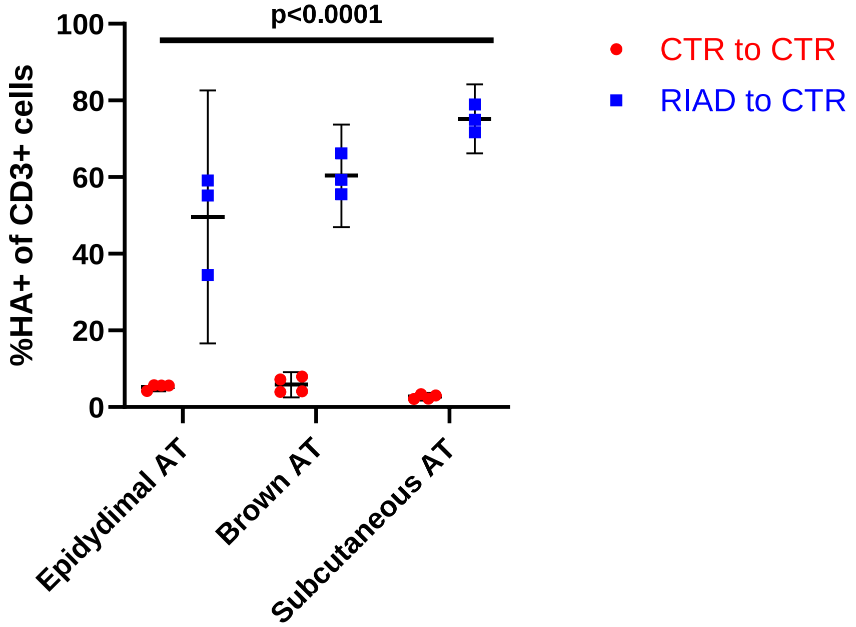

Figure S8. HA tag positive CD3⁺ cells in adipose tissue of bone marrow recipients from transgenic and control mice, related to Figure 4.

Percentage HA tag positive CD3⁺ cells in epidydimal (left), subcutaneous (middle) and brown (right) adipose tissue. Adipose tissue compartments after transplantation of bone marrow from transgenic and control mice.

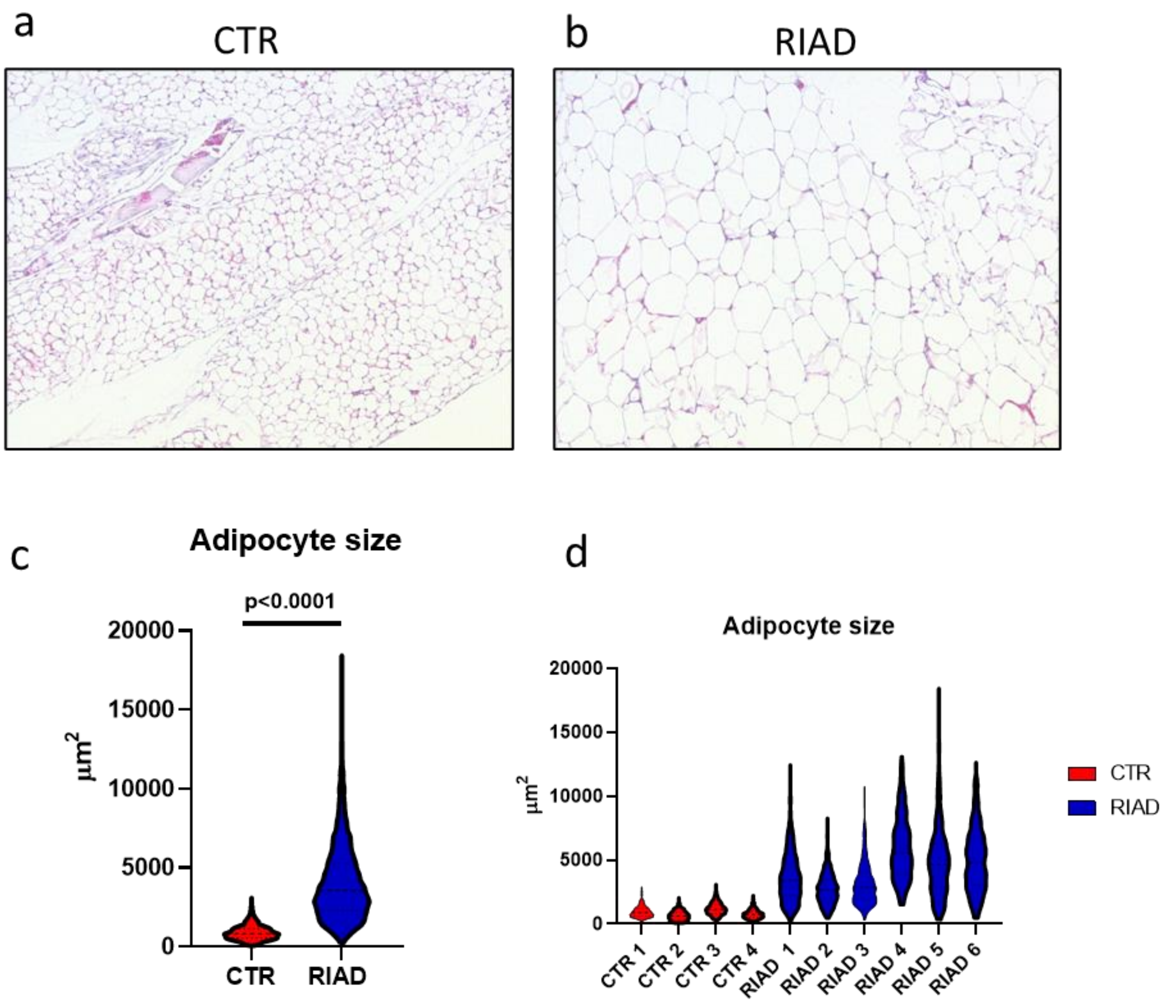

Figure S9. Adipocyte size in RIAD and control mice, related to Figure 6

Representative images of paraffin embedded visceral white adipose tissue from 6 months old female, stained with Haematoxylin and Eosin (HE) for **a)** control mice and **b)** RIAD mice. **c)** Violin plot of pooled adipocyte sizes (µm), according to genotypes (n=4-6), data analyzed with Mann Whitney test and **d)** violin plots of adipocyte sizes for each individual.

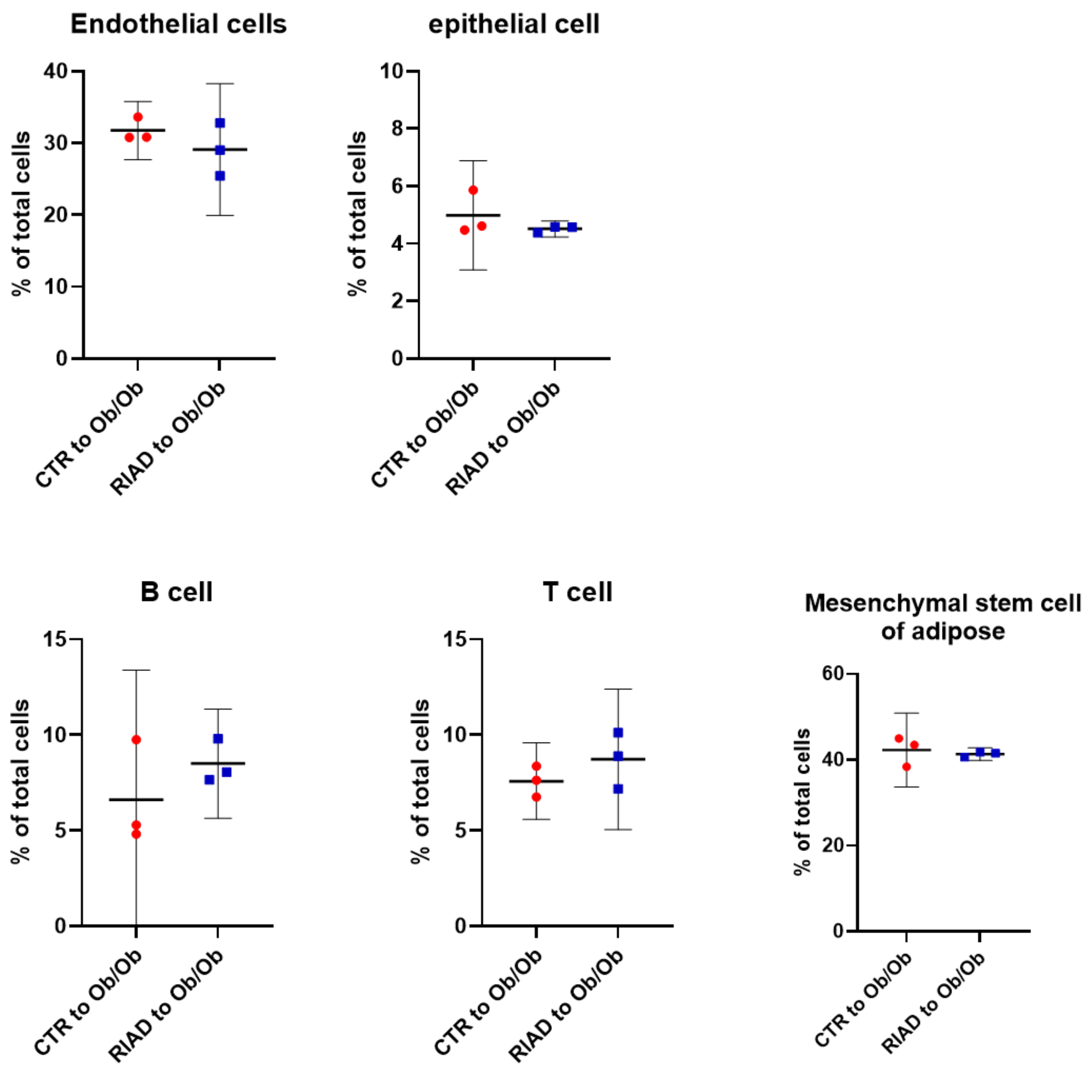

Figure S10. Estimated immune cell abundance in subcutaneous adipose tissue of RIAD and control mice, related to Figure 6.

Cell abundance in subcutaneous adipose tissue estimated by Cibersort digital cytomertry from same experiment shown in Figure 5B. Data presented as mean and 95% CI and analyzed with Mann Whitney test, n=3+3

Supplementary files overview

Supplementary file 1

Data related to Figure 2 and single-cell sequencing. Markers for PBMC cell clusters.

Supplementary file 2

Data related to Figure 2 and single-cell sequencing. Markers for T-cell clusters.

Supplementary file 3

Data related to Figure 2 and single-cell sequencing. DEGs; T-cell cluster 4 vs T-cell cluster 1 and T-cell cluster 4 vs T-cell cluster 2. Metascape-file included.

Supplementary file 4

Data related to Figure 6 and RNA-sequencing of white adipose tissue from bone marrow transplanted ob/ob mice vs control. Metascape-files.

Supplementary file 5

Data related to Figure 6 and RNA-sequencing of white adipose tissue from bone marrow transplanted ob/ob mice vs control. NicheNetR analysis finding potential upstream regulators for genes enriched in top 5 terms or pathways.
